# Inducing vulnerability to InhA inhibition restores isoniazid susceptibility in drug-resistant *Mycobacterium tuberculosis*

**DOI:** 10.1128/mbio.02968-23

**Published:** 2024-01-31

**Authors:** Gregory A. Harrison, Erin R. Wang, Kevin Cho, Yassin Mreyoud, Souvik Sarkar, Fredrik Almqvist, Gary J. Patti, Christina L. Stallings

**Affiliations:** 1 Department of Molecular Microbiology, Center for Women’s Infectious Disease Research, Washington University School of Medicine, St. Louis, Missouri, USA; 2 Department of Chemistry, Washington University in St. Louis, St. Louis, Missouri, USA; 3 Department of Medicine, Washington University School of Medicine, St. Louis, Missouri, USA; 4 Center for Metabolomics and Isotope Tracing, Washington University in St. Louis, St. Louis, Missouri, USA; 5 Department of Chemistry, Umeå University, Umeå, Sweden; 6 Umeå Centre for Microbial Research, UCMR, Umeå University, Umeå, Sweden; New York University School of Medicine, New York, New York, USA; University of Minnesota, Minneapolis, Minnesota, USA

**Keywords:** *Mycobacterium tuberculosis*, antibiotic resistance, isoniazid, mycolic acids, KatG

## Abstract

Of the approximately 10 million cases of *Mycobacterium tuberculosis* (*Mtb*) infections each year, over 10% are resistant to the frontline antibiotic isoniazid (INH). INH resistance is predominantly caused by mutations that decrease the activity of the bacterial enzyme KatG, which mediates the conversion of the pro-drug INH to its active form INH-NAD. We previously discovered an inhibitor of *Mtb* respiration, C10, that enhances the bactericidal activity of INH, prevents the emergence of INH-resistant mutants, and re-sensitizes a collection of INH-resistant mutants to INH through an unknown mechanism. To investigate the mechanism of action of C10, we exploited the toxicity of high concentrations of C10 to select for resistant mutants. We discovered two mutations that confer resistance to the disruption of energy metabolism and allow for the growth of *Mtb* in high C10 concentrations, indicating that growth inhibition by C10 is associated with inhibition of respiration. Using these mutants as well as direct inhibitors of the *Mtb* electron transport chain, we provide evidence that inhibition of energy metabolism by C10 is neither sufficient nor necessary to potentiate killing by INH. Instead, we find that C10 acts downstream of INH-NAD synthesis, causing *Mtb* to become particularly sensitive to inhibition of the INH-NAD target, InhA, without changing the concentration of INH-NAD or the activity of InhA, the two predominant mechanisms of potentiating INH. Our studies revealed that there exists a vulnerability in *Mtb* that can be exploited to render *Mtb* sensitive to otherwise subinhibitory concentrations of InhA inhibitor.

**IMPORTANCE:**

Isoniazid (INH) is a critical frontline antibiotic to treat *Mycobacterium tuberculosis* (*Mtb*) infections. INH efficacy is limited by its suboptimal penetration of the *Mtb*-containing lesion and by the prevalence of clinical INH resistance. We previously discovered a compound, C10, that enhances the bactericidal activity of INH, prevents the emergence of INH-resistant mutants, and re-sensitizes a set of INH-resistant mutants to INH. Resistance is typically mediated by *katG* mutations that decrease the activation of INH, which is required for INH to inhibit the essential enzyme InhA. Our current work demonstrates that C10 re-sensitizes INH-resistant *katG*-hypomorphs without enhancing the activation of INH. We furthermore show that C10 causes *Mtb* to become particularly vulnerable to InhA inhibition without compromising InhA activity on its own. Therefore, C10 represents a novel strategy to curtail the development of INH resistance and to sensitize *Mtb* to sub-lethal doses of INH, such as those achieved at the infection site.

## INTRODUCTION

The disease tuberculosis (TB), caused by *Mycobacterium tuberculosis* (*Mtb*), remains a global health threat. As of 2019, TB was reported to be the 13th leading cause of death worldwide, and the current COVID-19 pandemic has exacerbated challenges in TB disease surveillance and global control efforts (1, 2). A major obstacle in the treatment of *Mtb* infections is that the sterilizing activity of antibiotics is slow and sometimes incomplete at the site of infection due to several contributing factors. The penetration of antibiotics into the *Mtb* lesion can be limited, causing *Mtb* to be exposed to fluctuating and often subinhibitory concentrations of antibiotics (3). In addition, *Mtb* has the propensity to develop phenotypically drug-tolerant populations in the host, which allows a population of *Mtb* to persist despite exposure to antibiotics (3–6). Long treatment regimens are required to overcome the issues of drug penetration and bacterial drug tolerance to ultimately clear the infection. The standard of care for the treatment of active TB lasts 6 months, including a 2-month intensive phase of regular doses of isoniazid (INH), rifampicin, pyrazinamide, and ethambutol (EMB) followed by a 4-month continuation phase of INH and rifampicin (7). Recently, the World Health Organization approved the recommendation for a shortened 4-month treatment regimen that can be made available to some patients, which includes a 2-month intensive phase of INH, rifapentine, pyrazinamide, and moxifloxacin, followed by 2 months and 1 week of INH, rifapentine, and moxifloxacin (7). As a component of the intensive and continuation phases of both longer and shorter treatment regimens, INH is a critical frontline antibiotic that is the cornerstone of our current anti-TB regimens.

The utility of INH for the treatment of *Mtb* infections is threatened by the emergence and prevalence of INH-resistant mutant strains of *Mtb*. An estimated 10.7% of newly infected and 27.2% of previously treated cases are INH-resistant (8). INH is a prodrug, and resistance is most commonly caused by mutations in the gene *katG*, which encodes the sole bifunctional catalase-peroxidase enzyme in *Mtb* that is also responsible for converting INH to its active form within the bacteria (8–10). KatG is an oxidative defense enzyme, and its typical substrates are H_2_O_2_ and other peroxides (11). However, KatG acts on INH as a non-canonical substrate to generate a radical intermediate of INH (12), which spontaneously reacts with and attaches to the abundant cofactor NAD(H), forming INH-NAD (10, 13). The INH-NAD adduct, the activated form of INH, inhibits the enzyme InhA (10, 13, 14), which is the enoyl-acyl carrier protein reductase enzyme that functions in the fatty acid synthase II (FAS-II) system (15, 16). InhA is required for the FAS-II system to elongate the shorter fatty acids synthesized by FAS-I to generate long lipid precursors that are subsequently converted to mycolic acids (MAs) through a multi-step process. MAs are an essential structural component of the outermost layer of the *Mtb* cell envelope and, therefore, by inhibiting InhA, INH-NAD compromises the integrity of the *Mtb* cell envelope, leading to growth inhibition and death (17, 18).

Identifying ways to enhance the antibacterial activity of INH has the potential to greatly improve the standard of care for TB. To this end, we recently reported the identification of the bicyclic 2-pyridone compound C10 as a potentiator of INH activity in *Mtb* (19). At concentrations that on their own do not inhibit growth, C10 promotes the killing of *Mtb* by INH and prevents the emergence of spontaneous INH-resistant mutants (19). Whereas high-level resistance to INH mediated by mutations in *katG* is generally considered to render INH ineffective (20), we discovered that C10 was able to re-sensitize multiple INH-resistant *katG* mutants to inhibition by INH, which had previously not been thought to be possible. The ability of C10 to potentiate INH activity in both WT and INH-resistant *katG* mutant strains of *Mtb* demonstrates that there is a vulnerability in the bacteria that can be exploited to enhance the antimicrobial activity of INH and even circumvent INH resistance (19). Understanding the target and mechanism of action of C10 could lead to the discovery of novel therapeutic approaches that can be used in the clinic to disarm INH resistance in *katG* mutants.

In previous work, we performed RNA-sequencing on C10-treated *Mtb* and found that C10 induces a transcriptional signature consistent with inhibition of respiration (19). We subsequently demonstrated that C10 blocked *Mtb* oxygen consumption and decreased bacterial ATP levels, suggesting that C10 disrupts *Mtb* energy metabolism (19). In the current study, we aimed to determine how C10 potentiates killing by INH and whether this potentiation is linked to inhibition of *Mtb* energy metabolism. We used a combination of forward genetics and chemical biology to reveal that while some of the effects of C10 are mediated through its impact on energy homeostasis, the inhibition of respiration by C10 is not required for C10 to potentiate INH, thereby uncoupling these two effects of C10 on *Mtb* physiology. Instead, we present evidence that C10 restores INH susceptibility in a subset of resistant mutants by enhancing the bacterial vulnerability to InhA inhibition. Our findings reveal potential strategies to improve the efficacy of INH as well as other antibiotics that target mycolic acid metabolism in *Mtb*.

## RESULTS

### Isolation of mutants that are resistant to C10 growth inhibition and ATP depletion

**Fig 1:**
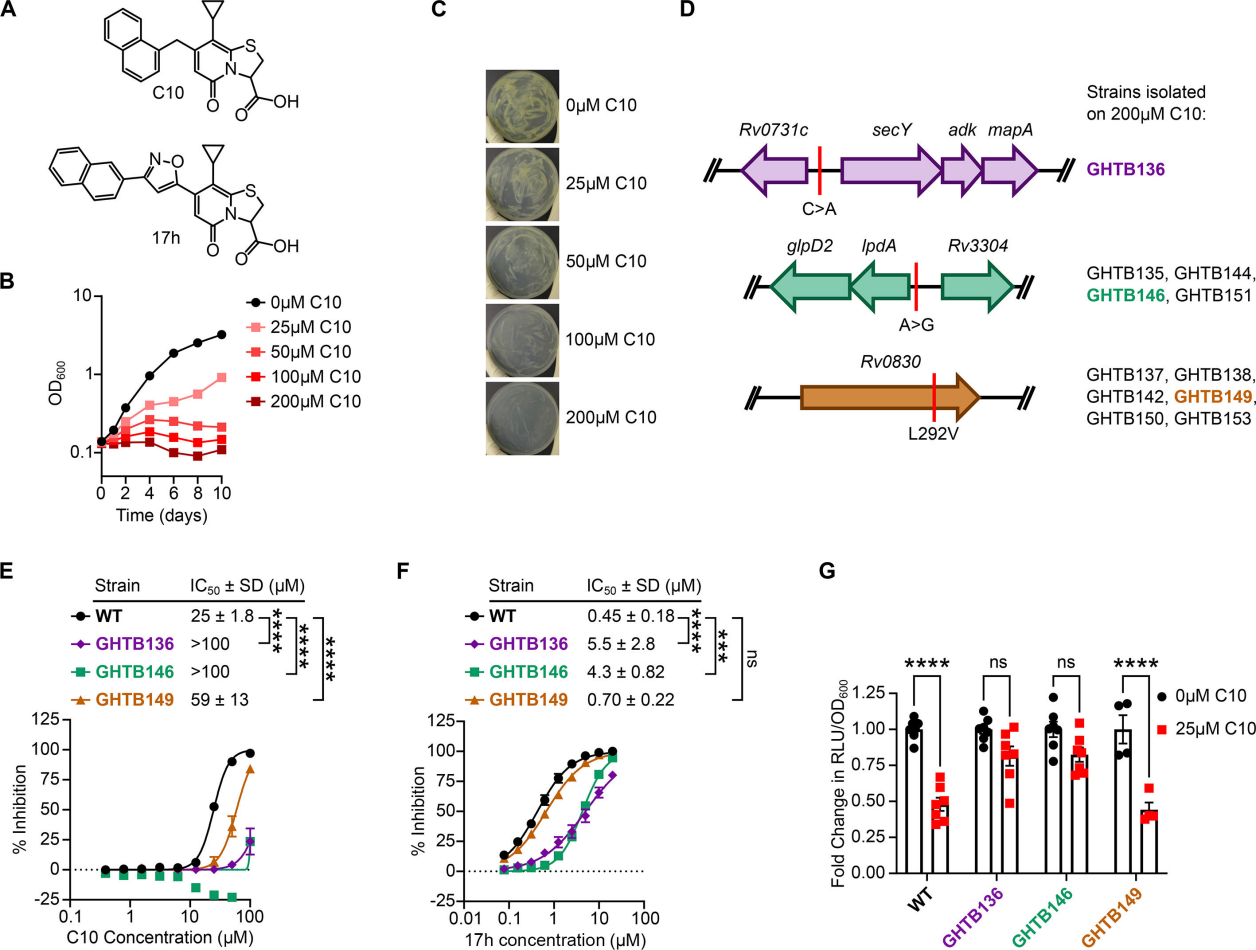
Isolation and characterization of C10-resistant mutants. (**A**) Chemical structures of C10 and 17h. (**B**) WT *Mtb* was cultured in Sauton’s medium containing the indicated concentration of C10, and growth was measured by OD_600_ over time. (**C**) WT *Mtb* was spread on Sauton’s agar medium containing the indicated concentration of C10 and incubated at 37°C for 3 weeks. (**D**) Whole-genome sequencing of 11 C10-resistant mutants revealed three groups of mutants. The mutant loci are depicted along with the resultant nucleotide or amino acid change, and the GHTB strain numbers indicate the mutant isolates that harbored the depicted mutation. Representative strains GHTB136, GHTB146, and GHTB149 were selected for follow-up studies. (**E and F**) The indicated strain of *Mtb* was cultured in the presence of increasing concentrations of (**E**) C10 or 17h (**F**) for 1 week, and the % inhibition of *Mtb* growth and metabolism was determined using the resazurin assay, *n* = 6. (**G**) The indicated strain of *Mtb* was cultured in Sauton’s liquid medium containing 0 or 25 µM C10 for 24 h before ATP levels were measured by the BacTiter Glo assay. The relative luminescence units (RLUs) were normalized to the optical density (OD_600_) of the culture to control for differences in cell density. Fold change in ATP levels were calculated relative to the 0 µM C10 control for each strain, *n* = 4–7. A one-way ANOVA (**E and F**) or a two-way ANOVA (**G**) with Tukey’s post test was performed to determine statistically significant differences across samples. IC_50_ values in panels E and F were log-transformed before statistical significance testing was performed. Selected comparisons are depicted in the figure. ns, not significant; ****P < 0.0001. For all pairwise comparisons, please see Table S1.

To better understand the mechanism of action of C10 (Fig. 1A), we chose a forward genetic approach and isolated mutants that are resistant to C10, with the goal of identifying mutations in genes linked to the mechanism of action of C10. Previous studies used 25 µM C10 to disrupt *Mtb* energy homeostasis and deplete bacterial ATP (19). However, 25 µM C10 only results in a modest decrease in *Mtb* growth (19). Therefore, to select for C10-resistant mutants, we first determined the concentration of C10 that was sufficient to inhibit the growth of wild-type (WT) *Mtb*. By increasing the C10 concentration above 25 µM, we found that C10 caused dose-dependent inhibition of *Mtb* growth both in liquid media and on agar plates (Fig. 1B and C), consistent with our previous studies (19). We found that 200 µM C10 completely inhibited *Mtb* growth in both conditions, so we used this concentration to select for resistant mutants. By spreading *Mtb* on agar containing 200 µM C10 and allowing these agar plates to incubate for a total of 9 weeks, we eventually observed the emergence of spontaneous resistant colonies. We isolated 11 resistant mutants and performed whole-genome sequencing to identify mutations that confer the C10 resistance. We found that each resistant strain harbored one of 3 different mutations (Fig. 1D). To probe how these mutations impact C10 sensitivity, we chose representative strains that each harbor one of these three mutations as the sole nucleotide change that could be identified by whole-genome sequencing. Strain GHTB136 harbors a C to A substitution in the intergenic region 69 base pairs (bp) from the start of the putative S-adenosylmethionine (SAM)-methyl transferase *Rv0731c* and 92 bp upstream of the *secY-adk-mapA* operon. To determine if this mutation impacts the expression level of the neighboring genes, we performed quantitative real-time PCR (qRT-PCR) and found that the GHTB136 strain exhibited >100-fold upregulation of the *Rv0731c* gene and 2- to 6-fold upregulation of the *secY-adk-mapA* operon compared to WT (Fig. S1A). The GHTB146 strain harbors an A to G substitution in the intergenic region 9 bp upstream of the *lpdA-glpD2* operon and >100 bp upstream from the uncharacterized gene *Rv3304*. The *Mtb* LpdA enzyme is a dehydrogenase that can oxidize or reduce NAD(H) or NADP(H) with concomitant oxidation or reduction of quinones (21). GlpD2 has not been characterized in *Mtb* but based on homology to the GlpD enzyme of *Escherichia coli* (22), it is predicted to be a glycerol-3-phosphate dehydrogenase that interconverts glycerol-3-phosphate and dihydroxyacetone phosphate with concomitant oxidation or reduction of menaquinone/menaquinol in the membrane. Since *lpdA* is a leaderless transcript (23), this mutation is likely located within the RNA polymerase binding region. Indeed, qRT-PCR analysis revealed that the GHTB146 mutant exhibited 4- to 8-fold upregulation of the *lpdA-glpD2* operon, but no change in expression of the *Rv3304* gene compared to WT (Fig. S1B), suggesting that this mutation enhances *lpdA-glpD2* promoter activity. The third representative strain, GHTB149, harbors a missense mutation within the putative SAM-methyl transferase *Rv0830* that results in the substitution of valine for a leucine residue at position 292 (L292V). Out of all of the genes identified by this approach, *secY*, *adk*, *mapA*, *lpdA*, and *glpD2* have been characterized in *Mtb* or *Escherichia coli*, and their functions are listed in Fig. S1C (21, 22, 24–26). However, *Rv0731c* and *Rv0830*, the putative SAM-methyltransferases affected in GHTB136 and GHTB149, remain hypothetical with a predicted catalytic activity but no characterized function in *Mtb* (Fig. S1C).

We quantified the level of C10 resistance in the GHTB136, GHTB146, and GHTB149 strains using a resazurin microplate assay. This assay takes advantage of the redox-sensitive dye resazurin, which is blue in its oxidized form but becomes reduced to the fluorescent pink product resorufin as a result of bacterial growth and metabolism. Therefore, fluorescence can be monitored as a proxy for bacterial growth and metabolism. C10 inhibits WT *Mtb* in this assay with a half-maximal inhibitory concentration (IC_50_) of 25 µM (Fig. 1E) (19). In contrast, the C10 IC_50_ in both GHTB136 and GHTB146 was >100 µM and the IC_50_ of C10 in GHTB149 was 59 µM (Fig. 1E). We also confirmed that both GHTB136 and GHTB146 exhibited high-level resistance to the recently published more potent C10 analog, 17h (Fig. 1A) (27), whereas GHTB149 was unable to significantly suppress the effects of 17h (Fig. 1F). Therefore, although all three mutants are resistant to C10, the mutations in GHTB136 and GHTB146 confer a higher level of resistance than the GHTB149 strain.

We previously showed that C10 inhibits respiration and depletes ATP levels in WT *Mtb* (19). Therefore, to directly determine how the mutations in the C10-resistant strains affected ATP-depletion by C10, we cultured WT, GHTB136, GHTB146, and GHTB149 in the presence and absence of 25 µM C10 for 24 h and quantified bacterial ATP levels using a luciferase-based BacTiter Glo assay (Fig. 1G). Similar to WT, the GHTB149 strain exhibited a decrease in bacterial ATP in response to C10, suggesting that the low level of resistance conferred by the *Rv0830* L292V mutation is not sufficient to overcome the depletion of ATP by C10. In contrast, C10 did not significantly decrease the ATP levels in the GHTB136 and GHTB146 strains (Fig. 1G), demonstrating that these mutants are resistant to the ATP-depleting effects of C10. Notably, none of the mutants exhibited an altered level of ATP at baseline (Fig. S1D), indicating that these strains do not overcome the effects of C10 by harboring increased pools of ATP but instead are able to maintain ATP levels during C10 treatment. Collectively these findings demonstrate that C10-mediated growth inhibition is linked to ATP depletion, as mutants that maintain ATP levels in the presence of C10 are able to overcome the toxicity of C10. To determine if the GHTB136 and GHTB146 strains are more generally resistant to ATP depletion, we quantified the sensitivity of the C10-resistant mutants to the ATP synthase inhibitor bedaquiline (BDQ) or the protonophore CCCP, compounds known to inhibit ATP synthesis by targeting the electron transport chain (ETC) (28, 29). We found that GHTB146 and GHTB149 exhibited a modest but significant 2.3- and 1.4-fold increase in the BDQ IC_50_, respectively (Fig. S1E), and GHTB136, GHTB146, and GHTB149 all exhibited a subtle but significant <1.5-fold increase in the CCCP IC_50_ compared to WT (Fig. S1F). While statistically significant, these minor changes in sensitivity to BDQ and CCCP indicate that GHTB136, GHTB146, and GHTB149 are especially resistant to the effects of C10, and the mechanism of resistance in these strains does not confer the same degree of cross-resistance to respiration inhibitors in general.

### The effects of C10 on ATP levels are associated with sensitization to the respiration inhibitor Q203 but not INH

**Fig 2:**
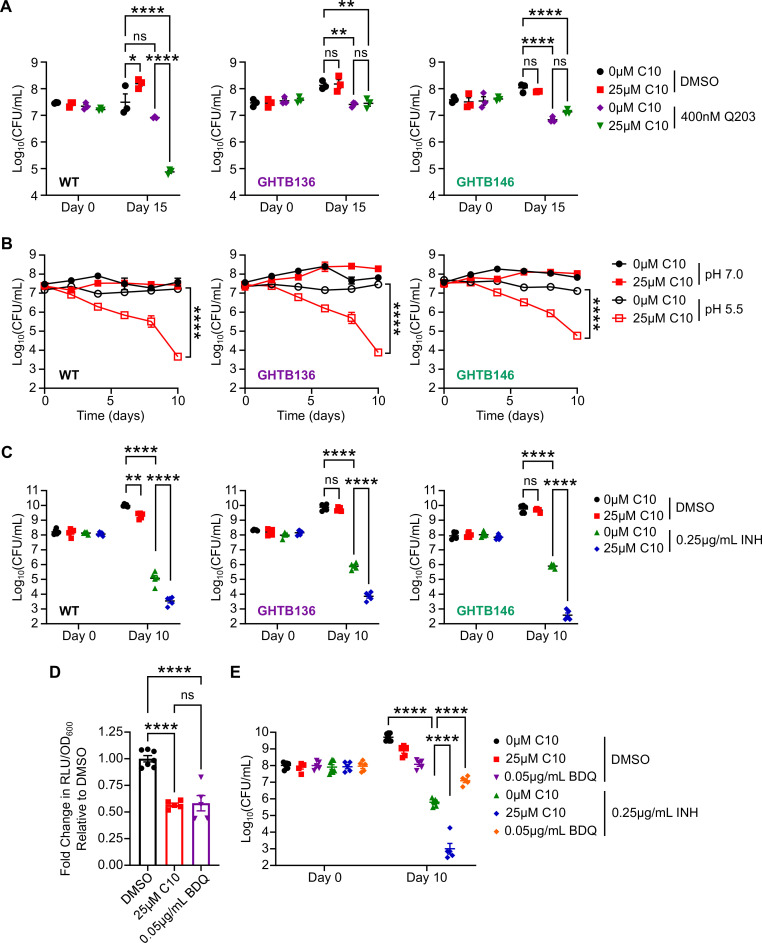
The effects of C10 on ATP levels are associated with sensitization to the respiration inhibitor Q203 but not INH. (**A**) WT, GHTB136, and GHTB146 *Mtb* were cultured in Sauton’s medium in the presence of the indicated concentration of C10 and Q203 and CFU/mL were enumerated on days 0 and 15 of the treatment, *n* = 3. (**B**) WT, GHTB136, and GHTB146 *Mtb* were cultured in Sauton’s medium adjusted to pH 7.0 (filled symbols) or pH 5.5 (open symbols) with or without 25 µM C10 and the CFU/mL were enumerated at the indicated time points, *n* = 3. (**C**) WT, GHTB136, or GHTB146 *Mtb* were cultured in Sauton’s liquid medium containing the indicated concentrations of C10 and INH and the CFU/mL was enumerated, *n* = 5. (**D**) WT *Mtb* was cultured in Sauton’s liquid medium containing 25 µM C10 or 0.05 µg/mL BDQ for 24 h before ATP levels were measured by the BacTiter Glo assay. The RLU were normalized to the OD_600_ of the culture to control for differences in cell density. Fold change in ATP levels were calculated relative to the DMSO control, *n* = 5–7. (**E**) WT *Mtb* was cultured in Sauton’s liquid medium containing the indicated concentrations of C10, BDQ, and/or INH, and the CFU/mL was enumerated, *n* = 5–7. A two-way ANOVA with Tukey’s post test was performed to determine statistically significant differences across samples. Selected comparisons are depicted in the figure. ns. not significant; *P < 0.05; **P < 0.01; and ****P < 0.0001. For all pairwise comparisons, please see Table S1.

Since the GHTB149 mutant only had low-level resistance to C10 (Fig. 1E), was not cross-resistant to the more potent C10 analog 17h (Fig. 1F), and was not resistant to the ATP-depleting effects of C10 (Fig. 1G), we focused our subsequent analysis on GHTB136 and GHTB146. In addition to decreasing ATP levels, C10 treatment potentiates the bactericidal effects of the cytochrome *bc*_1_ inhibitor Q203, low pH, and INH in WT *Mtb* (19). Therefore, we sought to determine if the mutations in GHTB136 and GHTB146 affected these additional consequences of C10 treatment. In particular, for Q203, we hypothesized that the ability of C10 to potentiate killing by Q203 was dependent on its effect on *Mtb* energy metabolism because respiration inhibitors often exhibit synergistic activity with other inhibitors of bacterial respiration (30–32). Indeed, when we exposed GHTB136 and GHTB146 to C10 and/or Q203 and monitored survival after 15 days of treatment, we found that C10 was unable to potentiate killing of GHTB136 and GHTB146 by Q203 (Fig. 2A). This is in contrast to WT *Mtb*, where C10 in combination with Q203 causes a significant 2 orders of magnitude decrease in the number of colony forming units (CFUs)/mL compared to Q203 alone (Fig. 2A) (19). These findings support that the ability of C10 to disrupt *Mtb* energy homeostasis is required for C10 to potentiate the bactericidal activity of Q203. Sensitivity to low pH is also a phenotype that is associated with inhibition of mycobacterial energy homeostasis (33, 34). However, when we examined the effect of C10 on GHTB136 and GHTB146 survival in pH 5.5 media, we found that C10 still mediated decreased survival of GHTB136 and GHTB146 *Mtb* strains in low pH media, to a similar magnitude as WT *Mtb* (Fig. 2B). These data suggest that the mechanisms that allow GHTB136 and GHTB146 to resist C10-mediated growth inhibition in pH 7.0 media are not sufficient to resist killing by C10 in low pH media.

In addition to its effects on energy homeostasis, we previously showed that C10 potentiates killing by INH (19). However, whether the disruption of energy homeostasis by C10 contributes to its ability to enhance the bactericidal activity of INH remains unknown. We used GHTB136 and GHTB146 to test if disruption of energy homeostasis is required for C10 to potentiate INH by culturing WT *Mtb*, GHTB136, and GHTB146 in media containing 25 µM C10 and/or 0.25 µg/mL INH and enumerating CFU/mL after 10 days of treatment to determine the number of viable bacteria. Similar to our previously reported results, C10 enhanced the bactericidal effect of INH against WT *Mtb*, leading to approximately two orders of magnitude fewer CFU/mL after 10 days of treatment compared to INH alone (Fig. 2C) (19). In addition, we found that C10 still potentiated the bactericidal activity of INH against the GHTB136 and GHTB146 strains to a similar or even greater extent as compared to WT (Fig. 2C). Therefore, C10 can enhance killing by INH in strains that maintain ATP levels during exposure to C10, demonstrating that ATP depletion is not required for C10 to potentiate INH. In support of this finding, when we examined whether the direct ATP synthase inhibitor BDQ could recapitulate the effect of C10 on INH sensitivity, we found that depletion of bacterial ATP with BDQ did not potentiate killing by INH (Fig. 2D and E). Instead, BDQ in combination with INH resulted in one to two orders of magnitude increase in the number of viable bacteria compared to INH alone. These findings are consistent with previous reports that BDQ and other ETC inhibitors, including Q203 and CCCP, antagonize killing by INH (35–37). Depletion of ATP is, therefore, not sufficient to potentiate INH activity in *Mtb*.

### Isolation of mutants that are resistant to the combination of C10 and INH

Our findings that C10 still mediates bacterial killing in low pH and potentiates the bactericidal activity of INH in GHTB136 and GHTB146 (Fig. 2B–C) supports that the mutations in these C10-resistant strains specifically overcome the effects of C10 on energy homeostasis but are unlikely to represent the direct targets of C10. These findings also imply that C10 must impart another effect on *Mtb* that is not reversed in the GHTB136 and GHTB146 mutants to elicit the increased sensitivity to low pH and to INH. To specifically address how C10 potentiates INH activity in *Mtb*, we sought to identify genes that are required for C10 to potentiate INH by selecting for spontaneous *Mtb* mutants that can grow in the presence of C10 and INH. We had previously been unable to select for mutants that grew in the presence of 25 µM C10 and 0.5 µg/mL INH (19). Therefore, we decreased the selective pressure by lowering the concentration of INH. We inoculated WT *Mtb* onto agar media containing 25 µM C10 and 0.2 µg/mL INH, and incubated the bacteria for 4 months at 37°C. We isolated three spontaneous mutants that could grow on agar media containing 25 µM C10 and 0.2 µg/mL INH and performed whole-genome sequencing to identify the genetic basis for resistance. Two of the mutant strains harbored large genomic deletions. One strain, GHTB089, was deleted for 27.9 kilobases (kb) of its genome (Δ2145809–2173696), which disrupted 27 annotated genes, including deleting the first 1183 bp of *katG*. The second isolate, GHTB092, was deleted for 38.6 kb of its genome (Δ2132215–2170824), which included the entire *katG* gene and 36 additional annotated genes. In contrast, the third strain harbored a single point mutation when compared to the parental WT strain, a nucleotide change in *katG* that results in an early stop codon at tryptophan 198. We designated this strain *katG*^W198*^.

**Fig 3:**
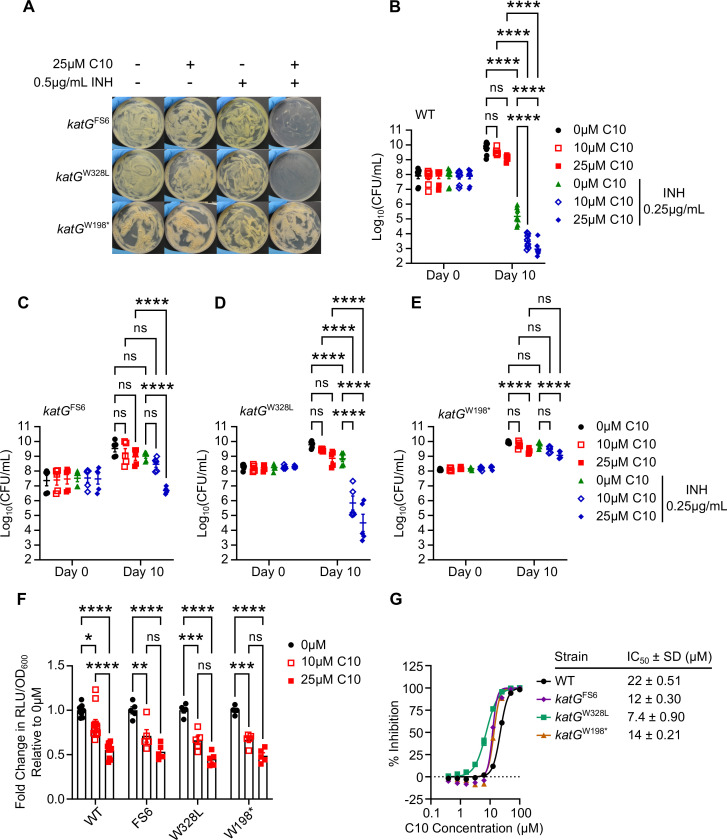
Forward genetic selection results in isolation of a *katG*^W198*^ mutant that is resistant to INH even in the presence of C10. (**A**) The indicated strain of *Mtb* was spread on Sauton’s agar medium containing 25 µM C10 and/or 0.5 µg/mL INH and incubated at 37°C for 3 weeks. (**B**) WT, (**C**) *katG*^FS6^, (**D**) *katG*^W328L^, and (**E**) *katG*^W198*^ *Mtb* were cultured in Sauton’s liquid medium containing the indicated concentrations of C10 and INH, and the CFU/mL was enumerated. The legend in panel **B** applies to panels **B–E**. (**F**) *Mtb* harboring the indicated *katG* allele was cultured in Sauton’s liquid medium containing 0, 10, or 25 µM C10 for 24 h before ATP levels were measured by the BacTiter Glo assay. The RLU was normalized to the OD_600_ of the culture to control for differences in cell density. Fold change in ATP levels was calculated relative to the 0 µM C10 control for each strain, *n* = 5–11. The data in panel F are also represented in Fig. S2 to highlight statistical comparisons between strains. (**G**) The indicated strain of *Mtb* was cultured in the presence of increasing concentrations of C10 for 1 week, and the % inhibition of *Mtb* growth and metabolism was determined using the resazurin assay, *n* = 3. A two-way ANOVA with Tukey’s post test was performed to determine statistically significant differences across samples. Selected comparisons are depicted in the figure. ns, not significant; *P < 0.05; **P < 0.01; ***P < 0.001; and ****P < 0.0001. For all pairwise comparisons, please see Table S1.

KatG is a 740-amino acid protein with several residues that are critical for catalase-peroxidase activity, including a heme-coordinating histidine at position 270 and catalytic residues at R104, H108, and W321. The *katG*^W198*^ mutation is predicted to result in truncation of over two-thirds of the KatG protein, including several of these essential residues. The selection of the *katG*^W198*^ mutant on media containing INH and C10 was perplexing because in our earlier study, we had shown that C10 potentiates INH in multiple INH-resistant *katG* mutants, including a *katG* mutant that harbors a frameshift mutation at amino acid 6 that results in an early stop codon (*katG*^FS6^) and a *katG* mutant with a single amino acid substitution of a leucine for a tryptophan at position 328 (*katG*^W328L^) (19). To determine if the *katG*^W198*^ mutant was truly unique from the previously studied INH-resistant *katG* mutants, we compared the ability of the *katG*^W198*^, *katG*^FS6^, and *katG*^W328L^ mutants to grow on agar media containing 0.5 µg/mL INH and/or 25 µM C10 (Fig. 3A). As expected based on our previous data (19), the *katG*^FS6^ and *katG*^W328L^ mutants grow well in the presence of either C10 or INH alone, but are re-sensitized to INH in the presence of C10 such that their growth is inhibited on agar containing both C10 and INH together (Fig. 3A). In contrast, the *katG*^W198*^ mutant grew on agar containing C10 alone, INH alone, and the combination (Fig. 3A), demonstrating that this strain is resistant to INH even in the presence of C10.

We monitored the effects of C10 and INH on the viability of each *katG* mutant strain compared to WT by culturing the bacteria in liquid media with or without C10 and/or INH for 10 days and enumerating CFU (Fig. 3B through E). Similar to our previously reported results, while the *katG*^FS6^ mutant can grow in the presence of INH or C10 alone, 25 µM C10 in combination with INH caused a significant decrease in the number of viable bacteria compared to C10 or INH alone (Fig. 3C) (19). Similar to the *katG*^FS6^ mutant, the *katG*^W328L^ mutant can grow in the presence of INH or C10 alone, but in combination with INH, 10 or 25 µM C10 was able to decrease the number of CFU/mL two to four orders of magnitude below the inoculum (Fig. 3D), demonstrating that C10 restores the bactericidal activity of INH against this mutant. In contrast, the CFU/mL of the *katG*^W198*^ mutant increased from days 0 to 10 in cultures treated with C10 alone, INH alone, and the combination (Fig. 3E). We found that 25 µM C10 significantly decreased the CFU/mL on day 10 compared to the untreated controls, but this was not enhanced by the addition of INH, indicating that C10 inhibits the growth of the *katG*^W198*^ mutant but is unable to potentiate INH in this strain.

The ability of the *katG*^W198*^ mutant to grow in the presence of C10 and INH could be due to resistance to the C10/INH combination or resistance to C10 specifically. To determine if the *katG*^W198*^ mutant was resistant to C10 activity, we examined the effect of C10 treatment on ATP levels in the *katG*^FS6^, *katG*^W328L^, and *katG*^W198*^ mutants by treating with 10 µM or 25 µM C10 for 24 h and then measuring ATP levels using the luciferase-based BacTiter Glo assay. C10 treatment caused a similar dose-responsive decrease in ATP in WT *Mtb* and the *katG* mutants as compared to the DMSO-treated cultures (Fig. 3F; Fig. S2). C10 also inhibited the *katG* mutants to a similar or even greater extent compared to WT in the resazurin microplate assay, with the IC_50_ of C10 in the WT, *katG*^FS6^, *katG*^W328L^, and *katG*^W198*^ strains being 22 µM, 12 µM, 7.4 µM, and 14 µM, respectively (Fig. 3G). These findings demonstrate that C10 is still able to disrupt *Mtb* energy homeostasis in *katG*^W198*^ and, therefore, the loss of INH potentiation in *katG*^W198*^ is not due to insensitivity to C10 activity.

### KatG activity is required for C10 to enhance INH inhibitory activity in *Mtb*

**Fig 4:**
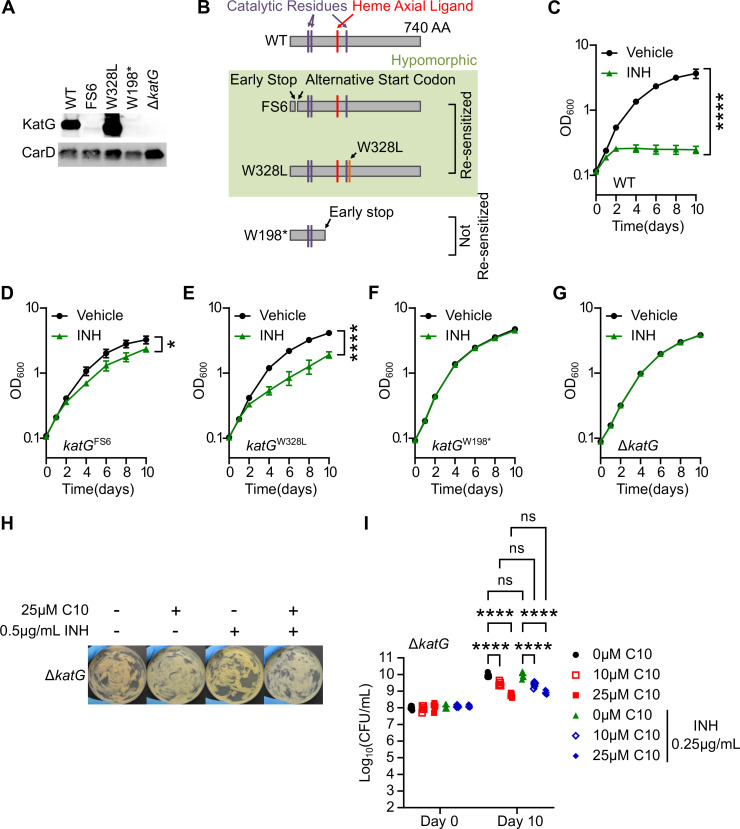
KatG activity is necessary for C10 to potentiate INH. (**A**) Western blot against whole-cell lysate from the indicated strain of *Mtb*. CarD was used as a loading control in this experiment. (**B**) Diagram of KatG protein indicating the required catalytic residues in the protein as well as the variant proteins expressed in each *katG* mutant. (**C–G**) The indicated strain of *Mtb* was treated with or without 0.25 µg/mL INH and growth was monitored over time by the optical density (OD_600_), *n* = 4. (**H**) Δ*katG Mtb* was spread on Sauton agar medium containing 25 µM C10 and/or 0.5 µg/mL INH and incubated at 37°C for 3 weeks before images were taken. (**I**) Δ*katG Mtb* was cultured in Sauton’s liquid medium containing 0, 10, or 25 µM C10 with or without 0.25 µg/mL INH and the CFU/mL were enumerated on 7H11 agar with no antibiotics to determine the number of viable bacteria in each culture at days 0 and 10 of treatment. A two-way ANOVA with Tukey’s post test was performed to determine statistically significant differences. ns, not significant; ****P < 0.0001. Relevant comparisons are depicted in the figure, but for all pairwise comparisons, please see Table S1.

Given the finding that C10 was able to re-sensitize both *katG*^FS6^ and *katG*^W328L^ mutant strains but not *katG*^W198*^ to INH, we hypothesized that there may be a functional difference between the KatG protein variant expressed in the *katG*^W198*^ mutant compared to *katG*^FS6^ and *katG*^W328L^. To begin to investigate this possibility, we probed the expression of KatG protein in each strain by western blot using a monoclonal α-KatG antibody (Fig. 4A). *katG*^W328L^ *Mtb* harbored similar or higher levels of KatG protein compared to WT *Mtb*, whereas *katG*^W198*^ *Mtb* did not express any full-length KatG protein, similar to a Δ*katG* strain. In contrast, the *katG*^FS6^ strain expressed a low abundance protein species that was recognized by the α-KatG antibody and migrated slightly faster than the full-length KatG protein expressed in WT *Mtb* (Fig. 4A). Upon closer examination of the *katG* mRNA sequence, we noted that there is a putative alternative GUG start codon downstream from the early stop codon introduced by the frameshift mutation in *katG*^FS6^ that may re-initiate translation in the correct frame beginning at codon 23. Therefore, we postulate that the faint band detected by western blot from *katG*^FS6^ cell lysate represents low-level expression of a variant KatG protein with a small N-terminal truncation (Δ1–22) that still contains all necessary catalytic residues (Fig. 4A and B).

Western blot analysis indicates that the *katG*^FS6^ and *katG*^W328L^ mutants express KatG protein variants that contain all necessary catalytic residues, while *katG*^W198*^ does not (Fig. 4A and B). To determine if the *katG*^FS6^ and *katG*^W328L^ mutants retained KatG catalytic activity, we cultured each strain in liquid media with or without 0.25 µg/mL INH and monitored the growth of the culture by measuring OD_600_ over time. We found that while WT *Mtb* was completely inhibited by 0.25 µg/mL INH (Fig. 4C), the *katG*^FS6^ and *katG*^W328L^ mutants were able to grow in the presence of INH, eventually reaching an OD_600_ over 10-fold above the inoculum (Fig. 4D and E). However, both mutants exhibited a significant decrease in the OD_600_ compared to the untreated controls, indicating that although the *katG*^FS6^ and *katG*^W328L^ mutants exhibit decreased sensitivity to INH, some KatG activity was retained to impart this modest growth inhibition in the presence of INH (Fig. 4D and E). In contrast, the *katG*^W198*^ and Δ*katG* mutants were completely resistant and grew uninhibited in the presence of INH (Fig. 4F and G), supporting that both of these mutations result in *katG*-null alleles. Therefore, the *katG*^FS6^ and *katG*^W328L^ mutants that are re-sensitized to INH by C10 are hypomorphic for *katG*, exhibiting decreased KatG activity leading to INH resistance, but retaining a residual level of KatG enzymatic activity (Fig. 4D and E). In contrast, the *katG*^W198*^ mutant that is not re-sensitized to INH by C10 exhibits no KatG activity (Fig. 4F), similar to a *katG*-null strain (Fig. 4G). Based on these data, we hypothesized that some KatG activity is required for C10 to enhance INH sensitivity. To test this hypothesis, we examined whether C10 could sensitize a Δ*katG* mutant to INH. Deletion of *katG* phenocopied the *katG*^W198*^ mutant and enabled *Mtb* to grow on agar and in liquid media containing both C10 and INH (Fig. 4H and I), demonstrating that some KatG expression and activity is required for C10-mediated sensitization to INH.

### C10 induces vulnerability to inhibition by INH without altering KatG activity or INH-NAD levels

**Fig 5:**
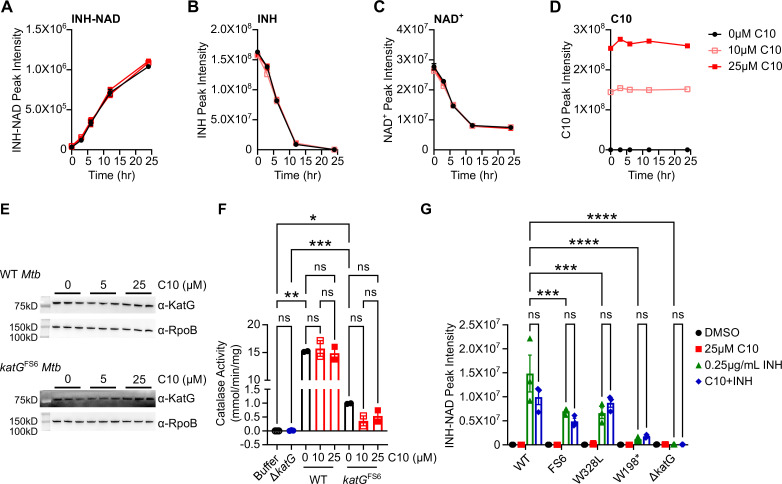
C10 enhances *Mtb* sensitivity to INH-NAD without changing its activation. (**A–D**) Purified KatG protein (200 nM) was incubated in 50 mM potassium phosphate buffer pH 7.0 containing INH, NAD^+^, and 50 µM MnCl_2_ with 0, 10, or 25 µM C10, and samples were taken at the indicated time points and analyzed via LC/MS. For each ion, the peak intensity was calculated as the area under the curve for (**A**) INH-NAD, (**B**) INH, (**C**) NAD^+^, and (**D**) C10, *n* = 3. The legend in panel D applies to panels A–D. (**E**) Either WT (top) or *katG*^FS6^ *Mtb* (bottom) was cultured in Sauton’s liquid medium in the presence of 0, 5, or 25 µM C10 for 3 days, and whole-cell lysate was collected for a Western blot using a monoclonal α-KatG antibody or an α-RpoB antibody as a loading control, *n* = 3. Note that the KatG blot from the *katG*^FS6^ samples has a high amount of background since the exposure time for this blot is substantially longer than for WT samples due to the very low level of KatG protein in this strain. (**F**) WT or *katG*^FS6^ *Mtb* was cultured in Sauton’s liquid medium in the presence of 0, 10, or 25 µM C10 for 6 days, and whole-cell lysate was collected to measure the catalase activity of the sample as a proxy for KatG activity, *n* = 2. Catalase activity is normalized to the amount of total protein in the whole-cell lysate sample. Note that lysate from the Δ*katG* mutant, *n* = 3, harbors no detectable amount of catalase activity compared to buffer alone, *n* = 3, demonstrating that the assay is specific for KatG. (**G**) *Mtb* with the indicated *katG* allele was cultured for 3 days in Sauton’s liquid medium in the presence and absence of 25 µM C10 and/or 0.25 µg/mL INH, after which polar metabolites were extracted from the culture and analyzed by LC/MS. The area under the curve was calculated by the integration of the peak to determine the peak intensity of INH-NAD in each bacterial sample, *n* = 3. Statistically significant differences were identified by Brown-Forsyth/Welch’s one-way ANOVA with Dunnet’s T3 post test in panel F and a two-way ANOVA with Tukey’s post test in panel G, and the relevant comparisons are indicated on the graph. ns, not significant; ***P < 0.001; ****P < 0.0001. For all pairwise comparisons, see Table S1.

Most strategies that renew the sensitivity of bacterial pathogens to antibiotics do so by increasing the levels of active antibiotics. Key examples of this are β-lactamase inhibitors that prevent the degradation of β-lactam antibiotics (38) and the recent discovery of SMARt-420, which increases the conversion of ethionamide (ETH) to its active form, ETH-NAD, in *Mtb* (39). In addition, *Mtb* encodes the enzyme CinA that cleaves NAD-drug adducts to promote tolerance to antibiotics like INH and ETH (40), highlighting that regulation of active drug concentration is a major mechanism of modulating drug efficacy. Therefore, we investigated whether C10 sensitizes *Mtb* to INH by promoting KatG activity to enhance the conversion of INH to INH-NAD, thus increasing the levels of INH-NAD in the cell. We monitored the effects of C10 on KatG enzymatic activity *in vitro* by incubating purified KatG protein with H_2_O_2_, a natural substrate of KatG, and monitoring KatG catalase activity as measured by the H_2_O_2_ degradation rate. We found that C10 did not enhance the rate or kinetics of H_2_O_2_ degradation by purified KatG protein *in vitro*, indicating that C10 did not directly affect KatG catalase activity (Fig. S3A). We next tested if C10 could specifically promote INH activation by purified KatG by monitoring the conversion of INH to INH-NAD *in vitro*. Previous work showed that INH activation by KatG occurred most efficiently in the presence of Mn^2+^ (10, 41). Therefore, we incubated KatG protein in buffer containing Mn^2+^ with INH and NAD^+^ in the presence or absence of C10 and monitored the levels of C10, INH, NAD^+^, and INH-NAD by liquid chromatography-mass spectrometry (LC-MS). We found that while the level of C10 remained unchanged over the course of the experiment, INH and NAD^+^ were depleted from the reaction with a concomitant increase in INH-NAD, as expected since INH and NAD^+^ are consumed to produce INH-NAD (Fig. 5A through D). C10 did not impact the rate or level of INH-NAD produced in these conditions. In addition, although we were able to detect the interaction between purified KatG and INH *in vitro* (Fig. S3B), we were unable to detect a direct interaction between C10 and KatG using a thermal shift assay (Fig. S3C), together supporting that C10 does not directly bind KatG or promote its enzymatic activity *in vitro*.

To determine if C10 affects KatG activity within the bacterium, we examined whether C10 promotes KatG expression, which could explain how C10 sensitizes WT and *katG* hypomorphic strains to INH but does not sensitize *katG*-null strains. We treated both WT and *katG*^FS6^ *Mtb* with C10 for 3 days and collected whole-cell lysate to perform western blot analysis for KatG. We found that C10 did not increase the protein levels of KatG in these conditions (Fig. 5E). We also monitored the effect of C10 on KatG catalase activity in *Mtb* by treating the bacteria with C10 for 6 days, collecting whole-cell lysate, and measuring the H_2_O_2_ degradation rate of the lysate. The Δ*katG* mutant exhibited no H_2_O_2_ degradation in this assay, demonstrating that the assay is specific for KatG (Fig. 5F). While the lysate from *katG*^FS6^ *Mtb* had significantly more activity than the Δ*katG* mutant, this strain exhibited a greater than 10-fold reduction in catalase activity compared to WT, consistent with the *katG*^FS6^ strain being hypomorphic for *katG*. We found that C10 did not change the H_2_O_2_ degradation rate of WT or *katG*^FS6^ *Mtb* (Fig. 5F), confirming that C10 treatment does not enhance the expression or activity level of KatG within *Mtb*. However, this finding did not rule out whether C10 could enhance the levels of INH-NAD within the bacteria without affecting KatG activity. To determine if C10 promotes INH-NAD accumulation in *Mtb*, we cultured WT, *katG*^FS6^, and *katG*^W328L^ *Mtb* with and without C10 and/or INH for 3 days, collected aqueous metabolite extracts from the bacteria, and measured the amount of activated INH-NAD in the bacterial extract using LC-MS. We included the *katG*-null mutants *katG*^W198*^ and Δ*katG* as controls. We found that upon treatment with INH alone, WT, *katG*^FS6^, and *katG*^W328L^ strains all accumulated INH-NAD, and the *katG*^FS6^ and *katG*^W328L^ mutants produced significantly decreased levels of INH-NAD compared to WT *Mtb*, confirming that these strains are indeed hypomorphic for *katG* (Fig. 5G). As expected, the *katG*-null mutants *katG*^W198*^ and Δ*katG* were deficient in INH-NAD synthesis (Fig. 5G). C10 did not significantly increase the amount of INH-NAD in any of the strains tested (Fig. 5G) and, therefore, does not affect INH-NAD synthesis or degradation. These data demonstrate that C10 sensitizes *Mtb* to INH through a novel mechanism of action without impacting the levels of INH-NAD in the bacteria.

### C10 sensitizes *Mtb* to direct inhibition of InhA

**Fig 6:**
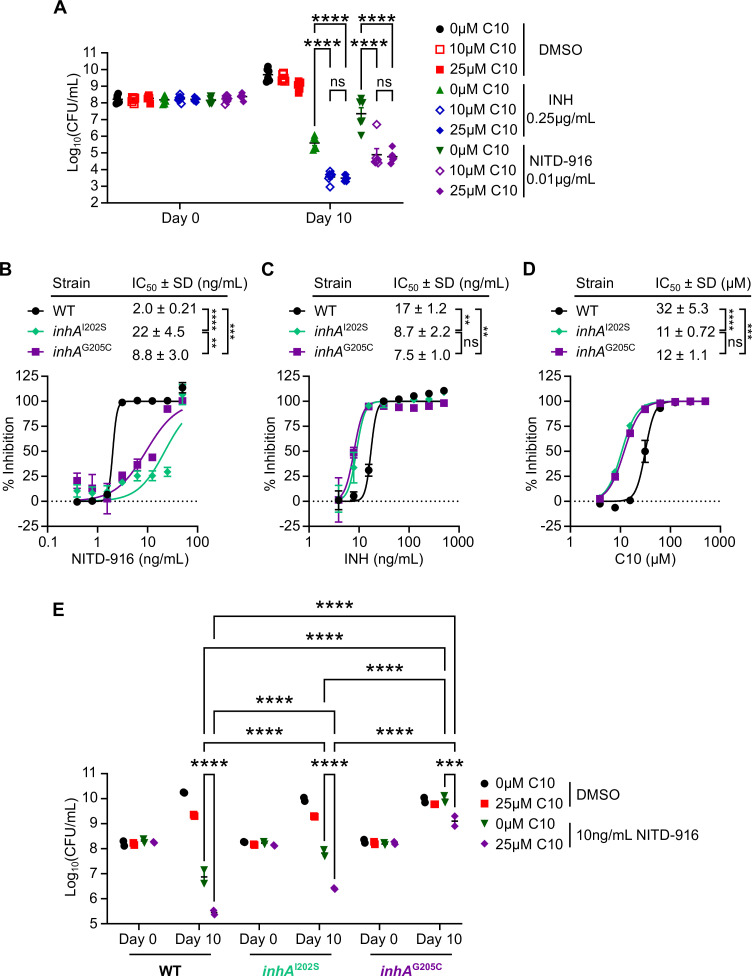
C10 enhances the vulnerability of *Mtb* to direct InhA inhibition. (**A**) WT *Mtb* was cultured in Sauton’s liquid medium with the indicated concentrations of C10, INH, and/or NITD-916, and CFU/mL was enumerated on days 0 and 10 of treatment to determine the number of viable bacteria in each sample, *n* = 6. (**B and C**) WT, *inhA*^I202S^, or *inhA*^G205C^ *Mtb* was exposed to increasing concentrations of (**B**) NITD-916 or (**C**) INH for 1 week and OD_600_ was quantified as a measure of growth. The % inhibition was quantified relative to untreated controls (100% growth) and streptomycin-treated controls (0% growth) for each strain. (**D**) WT, *inhA*^I202S^, or *inhA*^G205C^ *Mtb* was exposed to increasing concentrations of C10 and the % inhibition of *Mtb* growth and metabolism was determined using the resazurin assay, *n* = 3. Best fit curves were determined in GraphPad Prism. (**E**) WT, *inhA*^I202S^, or *inhA*^G205C^ *Mtb* was exposed to 25 µM C10 and/or 10 ng/mL NITD-916 and CFU/mL was enumerated on days 0 and 10 of the treatment. A two-way ANOVA with Tukey’s post test was used to identify statistically significant differences in panels A and E. IC_50_ values were log-transformed before performing a one-way ANOVA with Tukey’s post test to identify statistically significant differences in panels B–D. Selected comparisons are shown. ns, not significant; **P < 0.01; ***P < 0.001; ****P < 0.0001. For all pairwise comparisons, see Table S1.

Our data indicate that although the effect of C10 on INH sensitivity relies on KatG activity, C10 does not increase the expression or enzymatic activity of KatG. Based on these findings, we hypothesized that C10 acts downstream of KatG to enhance the antibacterial activity of INH-NAD after it is produced, such that even the *katG* hypomorphic strains that produce lower levels of INH-NAD become inhibited by this lower concentration in the presence of C10. INH-NAD inhibits *Mtb* growth by binding the NADH binding pocket in the enoyl-acyl carrier protein reductase InhA, leading to inhibition of mycolic acid biosynthesis (13, 14, 18, 42, 43). Therefore, it is possible that C10 renders *Mtb* more sensitive to inhibition of InhA. To test this possibility, we cultured WT *Mtb* with C10 and/or NITD-916, a direct inhibitor of InhA that does not require KatG or any other known enzyme for activation (44), and quantified the surviving CFU after 10 days of treatment. Similar to the effect of C10 on INH sensitivity, we found that treating *Mtb* with C10 in combination with NITD-916 caused a significant decrease in the number of surviving bacteria compared to NITD-916 alone, leading to an additional 2–3 orders of magnitude decrease in survival (Fig. 6A). Therefore, C10 increases *Mtb* sensitivity to direct InhA inhibition.

Given the ability of C10 to potentiate killing by NITD-916, we selected for mutants that could grow on agar containing both C10 and NITD-916 with the goal of identifying mutations in genes that are required for C10 to potentiate killing during direct InhA inhibition. We isolated two mutant colonies that emerged on agar containing both C10 and NITD-916 after 4 weeks and performed whole-genome sequencing. We found that each isolate harbored a mutation within the *inhA* gene that resulted in a single amino acid substitution, *inhA*^I202S^ and *inhA*^G205C^. Both the *inhA*^I202S^ and *inhA*^G205C^ mutants exhibited a significant increase in the IC_50_ for NITD-916 compared to WT *Mtb* in a microplate absorbance-based growth inhibition assay (Fig. 6B), confirming that they were more resistant to NITD-916. In contrast, the *inhA*^I202S^ and *inhA*^G205C^ mutants were significantly more sensitive to INH or C10 alone (Fig. 6C and D). When we cultured the *inhA*^I202S^ and *inhA*^G205C^ mutants in the presence of 10 ng/mL NITD-916 and enumerated the number of surviving bacteria after 10 days of treatment, we observed that while both *inhA* mutants were resistant to NITD-916, the *inhA*^G205C^ mutant was significantly more resistant to killing by NITD-916 than *inhA*^I202S^ (Fig. 6E). In addition, the degree of resistance to NITD-916 alone correlated with the level of increased resistance to the combination of C10 and NITD-916, where the *inhA*^G205C^ mutant was significantly more resistant to the C10 and NITD-916 combination than the *inhA*^I202S^ mutant (Fig. 6E). These data support that the potentiation of NITD-916 by C10 occurs through InhA inhibition, where NITD-916-mediated inhibition of InhA is required for C10 to potentiate its bactericidal activity. Thus, these data support the model that C10 sensitizes *Mtb* to inhibition of InhA.

### C10 sensitizes *Mtb* to inhibition of InhA without inhibiting InhA itself

**Fig 7:**
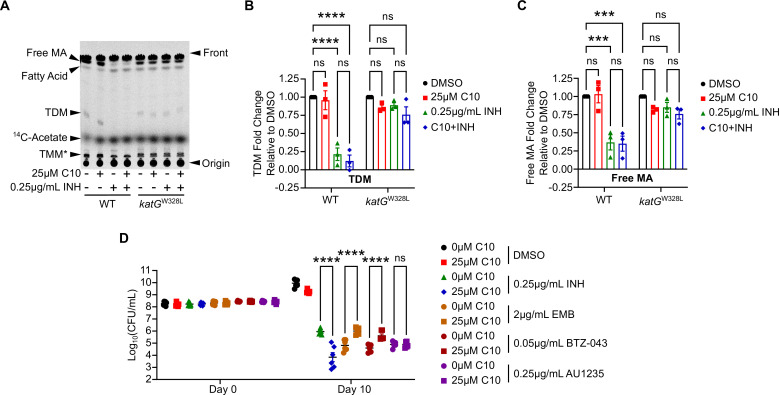
C10 sensitizes *Mtb* to InhA inhibition without altering bacterial InhA activity or making *Mtb* generally susceptible to cell envelope inhibitors. (**A**) WT or *katG*^W328L^ *Mtb* was cultured in Sauton liquid medium, treated with 25 µM C10 and/or 0.25 µg/mL INH, and immediately exposed to 2 µCi/mL of ^14^C-labeled acetate. After 20 h, lipids were extracted and analyzed by TLC to measure the *de novo* synthesis of mycolic acids and other lipids. The TLC plate was developed with 75:10:1 Chloroform:Methanol:H_2_O and radioactivity was analyzed by phosphorimaging. Bands corresponding to free mycolic acid (MA), fatty acid, trehalose dimycolate (TDM), and ^14^C-acetate were identified by comigration with a standard. Trehalose monomycolate (TMM) is indicated with a * to emphasize that this lipid is putatively identified, and not correlated with a standard. The plate in panel **A** is representative of three biological replicates, and the standards and additional replicates are shown in Fig. S4. (**B and C**) The intensity of bands corresponding to (**B**) TDM or (**C**) free MA were quantified in ImageJ, and normalized to the DMSO sample, with each WT sample being normalized to WT DMSO and each *katG*^W328L^ sample being normalized to *katG*^W328L^ DMSO in order to compare across replicates on separate plates, *n* = 3. (**D**) WT *Mtb* was cultured in Sauton’s liquid medium with the indicated concentrations of C10, INH, EMB, BTZ-043, and/or AU1235, and CFU/mL was enumerated on days 0 and 10, *n* = 6. Statistically significant differences were determined by two-way ANOVA and selected pairwise comparisons are depicted in the figure. ns, not significant; ***P < 0.001; ****P < 0.0001. For all pairwise comparisons, see Table S1.

One possible mechanism by which C10 could enhance the susceptibility of *Mtb* to InhA inhibition would be to decrease the expression or activity of InhA, thereby lowering the amount of INH-NAD or NITD-916 required to inhibit this target. When we analyzed our previously published RNA-sequencing data from C10-treated cultures, we found that after treatment with 25 µM C10 for 48 h, *inhA* was expressed at 1.1-fold relative to the untreated control (19), demonstrating that C10 does not decrease *inhA* expression at the transcriptional level. We next examined whether C10 compromises InhA activity by culturing *Mtb* with ^14^C-acetate and measuring *de novo* mycolic acid biosynthesis by thin-layer chromatography (TLC). In the final steps of mycolic acid biosynthesis, the mycolic acid moiety is coupled to trehalose to form trehalose monomycolate (TMM), which can be transported out of the cell through the activity of the TMM flippase MmpL3 (45, 46). The mycolic acid can then be trans-esterified from TMM to the arabinose moieties of arabinogalactan to form the inner leaflet of the mycolic acid layer that is covalently attached to the underlying cell wall layers (47–49). Alternatively, the mycolic acid can be trans-esterified to a second TMM molecule to form trehalose dimycolate (TDM) (50), which comprises a major component of the freely associated lipid layer that is intercalated within the covalently attached mycolic acids. Free mycolic acid, TMM, and TDM are the primary forms of mycolic acid that are not covalently linked to the cell wall, making them readily extractable and easy to separate by TLC, so we focused our analysis on these mycolic acid species as a read-out for *de novo* biosynthesis. Cultures of WT and *katG*^W328L^ *Mtb* were labeled with ^14^C-acetate for 20 hours in the presence or absence of C10 and/or INH before extracting whole cell lipids and monitoring the incorporation of the ^14^C into mycolic acids by TLC (Fig. 7A through C; Fig. S4). As standards, we used TDM purified from H37Ra (Invivogen), free mycolic acid saponified and extracted from the H37Ra TDM, the representative fatty acid oleic acid, and free ^14^C-acetate. Although we did not have a standard for TMM, we identified a band that we predict corresponds to TMM in our samples because it migrated slower than TDM due to the overall increased polarity and based on the migration pattern reported in published studies (51).

As expected, INH treatment of WT *Mtb* decreased the intensity of bands corresponding to free mycolic acid, TDM, and TMM (Fig. 7A through C; Fig. S4A through C), and significantly increased the intensity of a band that co-migrates with oleic acid (Fig. S4B). We believe this oleic acid co-migrating band represents FAS-I-generated fatty acids that serve as substrates for mycolic acid biosynthesis and, thus, it is not surprising that inhibition of InhA would lead to their accumulation (15–17). In contrast, INH treatment of *katG*^W328L^ *Mtb* did not affect the levels of free mycolic acids, TDM, or TMM over the 20-h period, supporting less efficient InhA inhibition due to the mutation in *katG* and decreased INH-NAD levels (Fig. 7A through C; Fig. S4). Addition of 25 μΜ C10 on its own or in combination with INH did not decrease the synthesis of free mycolic acid, TDM, or TMM in the WT or *katG*^W328L^ *Mtb* (Fig. 7A through C; Fig. S4A through C). Since 0.25 µg/mL INH causes nearly complete inhibition of mycolic acid biosynthesis in WT *Mtb*, it may be difficult to detect a further decrease in synthesis with this concentration of INH. We titrated INH to identify a concentration that only caused partial inhibition of mycolic acid biosynthesis and found that 0.0625 µg/mL INH significantly decreased free mycolic acid, TDM, and TDM biosynthesis in WT *Mtb*, but to a lesser degree than 0.25 µg/mL INH (Fig. S4D through G). Using 0.0625 µg/mL INH, we again tested if C10 could enhance the ability of INH to block mycolic acid biosynthesis in WT *Mtb*. In addition to 25 µM C10, we also included 50 µM C10 to investigate if a higher concentration of C10 is required to observe effects on mycolic acids. While 25 µM C10 did not significantly decrease free mycolic acid, TDM, or TMM biosynthesis on its own, 50 µM C10 caused a subtle but significant decrease in the labeling of free mycolic acids and TDM and a significant increase in the labeling of TMM (Fig. S4E through G). However, 50 µM C10 is also growth inhibitory (Fig. 1B) and, therefore, the effects on mycolic acids at this concentration may merely reflect inhibition of *Mtb* growth. In addition, including 25 µM and 50 µM C10 in combination with 0.0625 µg/mL INH did not cause a further decrease in the ^14^C-labeling of free mycolic acid, TDM, or TMM in WT *Mtb* (Fig. S4D through G). Monitoring TDM, TMM, and free mycolic acids only reflects changes in non-covalently attached mycolic acids, but the cell wall also harbors mycolic acids that are covalently attached to the arabinogalactan. In addition, mycolic acids produced by *Mtb* belong to three classes depending on their modifications, α-, methoxy-, and keto-mycolic acids, the relative proportions of which can impact the *Mtb* cell surface and physiology (52–54). To account for the covalently attached mycolic acids and to examine whether C10 alters the relative amounts of α-, methoxy-, or keto-mycolic acids, we derivatized whole cell extracts of *Mtb* to generate mycolic acid methyl esters, which liberates the covalently attached mycolic acids and also facilitates the separation of α-, methoxy-, and keto-mycolic acids. We found that treating WT *Mtb* with 25 µM C10 for 48 h did not significantly alter the relative amounts of α-, methoxy-, or keto-mycolic acid species (Fig. S4H and I). These findings suggest that 25 μΜ C10 does not alter the mycolic acid profile of *Mtb*.

Since C10 enhances the bactericidal effect of direct InhA inhibition without enhancing the inhibition of mycolic acid biosynthesis in the presence of INH, we wondered whether the potentiating effects of C10 are specific for InhA inhibitors, or if C10 generally sensitizes *Mtb* to cell envelope targeting antibiotics. We examined whether C10 impacted the sensitivity of *Mtb* to killing by the arabinogalactan-targeting antibiotics EMB and BTZ-043 (55, 56) and the MmpL3 inhibitor AU1235 (45). In combination with 2 µg/mL EMB or 0.05 µg/mL BTZ-043, 25 µM C10 increased *Mtb* survival (Fig. 7D). The finding that C10 antagonizes killing by EMB and BTZ-043 could be explained by the effect of C10 on energy homeostasis, since ETC inhibitors BDQ and CCCP also antagonize the bactericidal activity of EMB in *Mycobacterium bovis* (35). Consistent with the previous reports in *M. bovis*, treating *Mtb* with BDQ in combination with EMB recapitulates the antagonistic effect we observed with C10 (Fig. S4J), demonstrating that ATP depletion is sufficient to antagonize killing by EMB. Treatment of *Mtb* with 25 µM C10 in combination with the MmpL3 inhibitor AU1235 did not impact the number of surviving bacteria as compared to treatment with 0.25 µg/mL AU1235 alone (Fig. 7D). Since AU1235 inhibits mycolic acid metabolism downstream of InhA by blocking export of TMM to the cell envelope, these data suggest that the effects of C10 may be specific for inhibitors of mycolic acid synthesis that act upstream of MmpL3. While these studies do not rule out that C10 impacts the mycobacterial cell envelope in other ways, our data indicate that C10 specifically potentiates the bactericidal effect of InhA inhibitors without causing general susceptibility to cell envelope inhibitors.

## DISCUSSION

C10 represents a new strategy to improve the efficacy of antibiotic therapy against TB, where it sensitizes *Mtb* to killing by the frontline antibiotic INH as well as the cytochrome *bc*_1_ inhibitor Q203 that is in development for use in the clinic (19, 57). However, the mechanism by which C10 potentiates the bactericidal effect of these compounds remained unknown. Using two mutants resistant to growth-inhibitory concentrations of C10, we determined that the ability of C10 to potentiate Q203 is linked to its effects on ATP homeostasis. In contrast, both C10-resistant mutants were still susceptible to C10-mediated INH potentiation, demonstrating that the mechanism of INH potentiation is not dependent on depletion of ATP by C10, uncoupling the effect of C10 on ATP production from the sensitization to INH. Therefore, these C10-resistant mutants allowed us to differentiate between the mechanisms underlying C10-mediated potentiation of INH and Q203. Through our work to understand the mode of action of C10, we have revealed that there exist novel mechanisms to enhance the bactericidal activity of existing antibiotics, highlighting the utility of using C10 as a chemical tool to dissect *Mtb* physiology and antibiotic susceptibility.

The mechanism by which the C10-resistant mutants suppressed the ATP-depleting effects of C10 and allowed for growth in the presence of C10 remains an open question. An intergenic mutation in one of the C10-resistant mutants results in >100-fold upregulation of expression of the predicted SAM-methyltransferase *Rv0731c* and >2-fold upregulation of the *secY-adk-mapA* operon, while an intergenic mutation in the other C10-resistant mutant results in >4-fold upregulation of the *lpdA-glpD2* operon. We also isolated a mutant with low-level C10 resistance that harbors a missense mutation in the predicted SAM-methyltransferase *Rv0830*. When we examine our previously published RNA-sequencing data set to determine if C10 causes dysregulated expression of any of these genes, we find that *Rv0830*, *adk*, and *mapA* are each significantly upregulated in response to 25 µM C10 by 1.64-fold, 1.55-fold, and 1.35-fold, respectively, whereas the other genes are unchanged (19). Therefore, these genes are part of the *Mtb* transcriptional response to C10, perhaps to compensate for the inhibition of energy metabolism pathways during exposure to C10. Further supporting that the genes affected in the C10-resistant mutants represent a compensatory mechanism *Mtb* employs during C10 treatment and not the direct C10 target, the C10-resistant mutants maintain sensitivity to C10-mediated bactericidal activity in the presence of low pH and INH. In addition, these findings suggest that it is unlikely that the mechanism of resistance to C10 in these strains is mediated by inactivation of C10 by the enzymes that are upregulated, since we would expect that mutations that enable *Mtb* to degrade C10 or otherwise decrease its concentration would lead to resistance to all of the effects of C10. Further work is required to understand how these genes are involved in the ability of C10 to decrease ATP levels and inhibit growth in *Mtb*.

Instead of being associated with C10-mediated decreases in ATP, our data support a model where C10 potentiates INH by making *Mtb* particularly vulnerable to the inhibition of InhA, even in the INH-resistant *katG* hypomorphs that accumulate a significantly lower concentration of INH-NAD. Our findings show that C10 sensitizes *Mtb* to INH without changing the concentration of INH-NAD or decreasing the activity of its target InhA, which are the two predominant mechanisms of potentiating INH reported in the literature thus far (40, 58–60). In contrast to the potentiation strategies that increase the INH-NAD concentration or decrease InhA expression, which would be specific for INH or InhA inhibitors, respectively, it remains possible that C10 could sensitize *Mtb* more generally to additional inhibitors of mycolic acid biosynthesis. We found that C10 did not potentiate killing by the MmpL3 inhibitor AU1235, but it still remains to be tested whether C10 affects the sensitivity of *Mtb* to compounds that block steps in mycolic acid biosynthesis other than InhA that are upstream of MmpL3. Mycolic acid biosynthesis is essential in mycobacteria and not conserved in non-actinobacteria or eukaryotes, making this process a very attractive target for the development of specific antimycobacterials to treat TB and increasing the value of understanding how C10 sensitizes *Mtb* to inhibition of InhA.

The clinical utility of INH is currently being threatened by the increasing rates of INH-resistant TB cases. While resistance to INH can occur through multiple mechanisms, the predominant cause of INH resistance is mutation of *katG*, which accounts for an estimated 78.6% of INH-resistant strains (8). Clinically, mutations in *katG* are considered to confer a high level of resistance, often necessitating the use of an alternative treatment regimen. However, the overwhelming majority of these *katG* mutations are not null alleles. The most common resistance variant is an S315T amino acid substitution in KatG that decreases the enzyme’s affinity for INH (8, 61, 62). The S315T mutation and other single amino acid substitutions that are commonly identified in resistant isolates significantly impair the ability of KatG to activate INH, but these mutations do not completely abolish INH-NAD synthesis by the KatG enzyme *in vitro* (62, 63). The complete inactivation of KatG is likely detrimental to *Mtb* survival in the host due to the role of KatG in the oxidative stress response, which could explain why the majority of INH-resistant clinical isolates harbor single amino acid substitutions as opposed to more deleterious mutations (64–66). We found that C10 selectively potentiates killing by INH in *katG* mutants that retain some KatG enzymatic activity, indicating that it is possible to rescue the utility of INH against most clinically relevant INH-resistant isolates since most isolates will retain the low level of KatG activity. While the precise mechanism by which C10 induces sensitivity to InhA inhibition remains unclear, by deciphering how C10 promotes susceptibility to INH and NITD-916, we will reveal cryptic vulnerabilities in *Mtb* that can be exploited to enhance our current antimicrobial regimen.

In addition to resistance, INH efficacy can be limited by subinhibitory concentrations of antibiotics at the site of infection. For instance, a clinical study that quantified the distribution of antibiotics across lung lesions from TB patients within 24 h of dosing showed that approximately 35% of lesions harbored sub-inhibitory concentrations of INH, likely due to a combination of drug diffusion and host metabolism (3). Therefore, the *Mtb* within these lesions likely experience fluctuating concentrations of antibiotics that can be sub-inhibitory. C10 represents a possible strategy to sensitize *Mtb* to even sub-inhibitory concentrations of antibiotic, suggesting that in addition to circumventing INH resistance, C10 could enhance the efficacy of INH at the site of infection, although this remains to be tested. During the course of infection, *Mtb* is exposed to a myriad of host-derived stresses, including hypoxia within *Mtb*-containing lesions in the lung (67), as well as low pH within phagolysosomes in infected phagocytes (68, 69). C10 mediates the killing of *Mtb* during low pH stress and more potent C10 analogs, such as 17 h, decrease *Mtb* growth in a cultured murine macrophage cell line (19, 27), providing further evidence that C10 sensitizes *Mtb* to intracellular host-derived stresses. Thus, a better understanding of C10’s mechanism of action could provide new opportunities for targeting *Mtb* at the site of infection.

## MATERIALS AND METHODS

### Bacterial strains and growth conditions

*Mtb* Erdman strains (Table 1) were inoculated from a freezer stock into Middlebrook 7H9 liquid medium supplemented with 60 µL/L oleic acid, 5 g/L BSA, 2 g/L dextrose, 0.003 g/L catalase (OADC), 0.5% glycerol, and 0.05% Tween 80 and cultured at 37°C. Actively growing *Mtb* was then inoculated into Sauton’s liquid medium [0.5 g/L KH_2_PO_4_, 0.5 g/L MgSO_4_, 4.0 g/L L-asparagine, 6% glycerol, 0.05 g/L ferric ammonium citrate, 2.0 g/L citric acid, and 0.01% (wt/vol) ZnSO_4_, pH 7.0] supplemented with 0.05% Tween 80 and grown to late-log phase before use in growth curve and survival experiments. The Δ*katG* strain was generated using specialized transduction with the temperature-sensitive phage phAE87 engineered to harbor sequence homologous to regions upstream (Erdman nucleotides 2144027–2144776) and downstream (Erdman nucleotides 2146989–2147705) of *katG* and selected on 50 µg/mL hygromycin as previously described (70). For *Mtb* growth and survival experiments, *Mtb* was inoculated into roller bottles containing Sauton’s medium supplemented with Tween 80 at a starting OD_600_ of 0.1, and growth was measured by OD_600_ and survival was monitored as CFU/mL. Viable CFU from bacterial cultures were enumerated on Middlebrook 7H11 agar medium supplemented with OADC and 0.5% glycerol and plates were incubated at 37C with 5% CO_2_ for 2–3 weeks. To select for mutants resistant to both C10 and INH, the equivalent of 0.5 mL of OD_600_ = 1.0 of *Mtb* growing in 7H9 + OADC media was spread on Sauton’s agar containing 25 µM C10 and 0.2 µg/mL INH and incubated at 37°C and 5% CO_2_ for 4 months. Isolated colonies were passaged on agar containing 25 µM C10 and 0.2 µg/mL INH to ensure that they were resistant before performing whole-genome sequencing. For agar growth assays, 0.003 g/L bovine catalase was included in the Sauton’s agar, and the equivalent of 2.5 mL of OD_600_ = 1.0 of *Mtb* was spread on the agar surface to enhance the reproducibility of *Mtb* growth on the Sauton’s agar plates.

**TABLE 1:** *Mtb* strains used in this study

Strain	Description	Source
WT	Erdman	
GHTB136	Erdman selected on 200 µM C10, harbors a C to A single nucleotide substitution 69 bp from *Rv0731c* and 92 bp from *secY*	This study
GHTB146	Erdman selected on 200 µM C10, harbors an A to G single nucleotide substitution 9 bp from *lpdA*	This study
GHTB149	Erdman selected on 200 µM C10, harbors a single nucleotide substitution in *Rv0830* resulting in a L292V amino acid change	This study
*katG* ^FS6^	Erdman selected on 0.5 µg/mL INH	(19)
*katG* ^W328L^	Erdman selected on 0.5 µg/mL INH	(19)
*katG* ^W198*^	Erdman selected on 0.2 µg/mL INH and 25 µM C10, harbors a C to T single nucleotide substitution at position 2146398	This study
GHTB089	Erdman selected on 0.2 µg/mL INH and 25 µM C10, harbors deletion of Erdman nucleotides 2145809–2173696	This study
GHTB092	Erdman selected on 0.2 µg/mL INH and 25 µM C10, harbors deletion of Erdman nucleotides 2132215–2170824	This study
Δ*katG*	Erdman mutant generated by specialized transduction, in which nucleotides 2144776–2146988 were replaced with a Hygromycin resistance marker via double homologous recombination	This study
*inhA* ^I202S^	Erdman selected on 10 ng/mL NITD-916 and 25 µM C10, harbors a T to G single nucleotide substitution in *inhA* resulting in a I202S amino acid change	This study
*inhA* ^G205C^	Erdman selected on 10 ng/mL NITD-916 and 25 µM C10, harbors a G to T single nucleotide substitution in *inhA* resulting in a G205C amino acid change	This study

### Whole-genome sequencing

Genomic DNA was isolated using cetyltrimethylammonium bromide-lysozyme lysis, followed by phenol-chloroform-isoamyl alcohol extraction and isopropanol precipitation, as previously described (71). Whole-genome sequencing was performed by use of an Illumina NovaSeq 6000. The identification of single nucleotide polymorphisms was done using SeqMan NGen software (DNASTAR). The genomes were assembled and compared to the genomic DNA from the WT parental control strain, using Integrative Genome Viewer to visualize and confirm changes within regions of interest (72).

### Preparation of compounds

C10 was synthesized using previously described methods (73, 74) and prepared as an imidazole salt as described previously (19). Stocks of C10-imidazole were resuspended in DMSO. INH (Sigma) was dissolved in water, and NITD-916, BDQ, EMB (Sigma), BTZ-043, and AU1235 (MedChem Express) were dissolved in DMSO. In all experiments, the concentration of both DMSO and imidazole was normalized across all samples to ensure that any differences were due to the effect of the indicated compounds and not due to DMSO or imidazole.

### Detection of ATP

*Mtb* growing in Sauton’s media was treated with the indicated concentration of C10 for 24 h before the OD_600_ of each culture was measured and samples were removed. *Mtb* samples were inactivated at >95°C for 20 min and stored at −20°C until analyzing the ATP levels using the BacTiter Glo assay (Promega) as previously described (19). Samples were diluted 1:10, then mixed 1:1 with the BacTiter Glo reagent in a white, opaque 96-well dish, and the luminescence was read on a Synergy HT plate reader with a 1-s integration. The relative luminescence units (RLUs) were calculated by subtracting the luminescence of a media-only control from the luminescence value of each sample. The RLU/OD_600_ was determined to account for differences in bacterial density, and the fold change in each sample was calculated relative to the average of the 0 µM C10 control from that experiment to facilitate the combining of multiple experiments onto a single graph.

### Resazurin IC_50_ assays

Logarithmically growing *Mtb* was inoculated into Sauton’s medium in 96-well plates with wells containing increasing concentrations of the indicated compounds. *Mtb* was inoculated at an OD_600_ of 0.0025 in 200  µL per well. The plates were incubated at 37°C in 5% CO_2_ for 1  week, at which point 32.5  µL of a mixture containing an 8:5 ratio of 0.6  mM resazurin (Sigma) dissolved in 1× phosphate-buffered saline (PBS) to 20% Tween 80 was added, and the production of fluorescent resorufin was measured on a Synergy HT plate reader with excitation λ_ex_ = 530 nm and emission λ_em_ = 590 nm after incubation at 37°C in 5% CO_2_ overnight. For each assay, medium alone served as a negative control, and untreated *Mtb* was included as a positive control. The percent inhibition was calculated as the {[(fluorescence of the positive control − fluorescence of the negative control) − (fluorescence of the sample − fluorescence of the negative control)]/(fluorescence of the positive control − fluorescence of the negative control)} × 100.

### Growth inhibition IC_50_ assays

Logarithmically growing *Mtb* was inoculated into Sauton’s medium in 96-well plates with wells containing increasing concentrations of the indicated compounds. *Mtb* was inoculated at an OD_600_ of 0.05 in 200 µL per well. The plates were incubated at 37°C in 5% CO_2_ for 1  week, and the absorbance at wavelength λ = 600 nm was measured on a Synergy HT plate reader. For each assay, wells containing media alone served as a blank, 400 µg/mL streptomycin was included as a negative control, and untreated *Mtb* was included as a positive control. The percent inhibition was calculated similarly to that described for the resazurin assay above.

### Quantitative reverse transcription PCR (qRT-PCR)

RNA was isolated from WT, GHTB136, and GHTB146 *Mtb* growing in Sauton’s medium using TRIzol, and purified by chloroform extraction followed by isopropanol precipitation. cDNA was prepared using the SuperScript III first-strand synthesis kit (Invitrogen), and qPCR was performed using an SYBR green kit (Bio-Rad) with gene-specific primers (Table 2) on a CFX96 Real-Time System (Bio-Rad). The relative expression of genes was calculated using the 2^−ΔΔCt^ method, with *sigA* serving as an internal reference control gene.

**TABLE 2:** Primers used in this study

Primer #	Primer name	Sequence
GHP102	*lpdA* qPCR FWD	ACCCGGAAACAACCCAAGTTAC
GHP103	*lpdA* qPCR REV	TGGCGTCGTCGAAGTCGATATG
GHP104	*glpD2* qPCR FWD	AGCCGCTCCTCGAAGATGTTCC
GHP105	*glpD2* qPCR REV	ACGGCAGCGGCTTGACCAAATG
GHP106	*Rv0731c* qPCR FWD	CTTGGCGTCCAGTGTGGGTTTG
GHP107	*Rv0731c* qPCR REV	ACTGGCCATGCGTACGAAGAAG
GHP140	*Rv3304* qPCR FWD	GCGGACGAGAAGAACCTTGAC
GHP141	*Rv3304* qPCR REV	GGCCATCACACCTAGATAGCG
GHP142	*secY* qPCR FWD	CTGGGCATCGTCATTCTCTAC
GHP143	*secY* qPCR REV	CCGGAGAACAGGTTGATCAG
GHP144	*adk* qPCR FWD	GGAAGCCAAACGCTACTTGGATG
GHP145	*adk* qPCR REV	CGTTCGAGCATCTCGTGAAG
GHP146	*mapA* qPCR FWD	GCGACAAGGGAATCGCTTCAG
GHP147	*mapA* qPCR REV	TCCCGAACGAGCGTCCATAAC
GHP001	*katG* DS FWD *Xma*I	GCCCGGGGTTGGCCACCTCCGTGTCGAGC
GHP002	*katG* DS REV *Xba*I	GTCTAGATGCGCTGATTCGGGTTGATCG
GHP003	*katG* US FWD *Hind*III	GAAGCTTCACAGCATTCCTTCCAGGAGTTGGTG
GHP004	*katG* US REV *Xho*I	GCTCGAGACCCTCTACCACCTTCCTGCC
GHP097	*katG* pGEX FWD *Eco*RI	GGAATTCGTGCCCGAGCAACACCCACCCATTAC
GHP098	*katG* pGEX REV *Not*I	GGCGGCCGCATCAGCGCACGTCGAACCTGTCGAG

### Western blot for KatG protein

*Mtb* samples were pelleted and resuspended in buffer containing 10 mM sodium phosphate pH 8.0, 150 mM NaCl, 2 mM EDTA, 1 mM PMSF, 0.1% NP-40, and a 1× protease inhibitor cocktail (Roche), then lysed by bead beating, and filtered two times through a 0.22-µm Spin-X column (Costar) to remove unlysed *Mtb*. SDS-polyacrylamide gel electrophoresis was performed and samples were transferred to a nitrocellulose membrane, after which KatG was detected using a mouse monoclonal α-KatG antibody used at 1:500 dilution (clone IT-57; BEI Resources). Either CarD or RpoB served as a loading control, using a mouse monoclonal α-CarD antibody at 1:2,000 dilution (clone 10F05; Memorial Sloan-Kettering Cancer Center) or a mouse monoclonal α-RpoB antibody at 1:1000 dilution (clone 8RB13; Neoclone). The membrane was probed with a goat anti-mouse antibody conjugated to horseradish peroxidase and bands were visualized using the Western Lighting Plus-ECL reagent (PerkinElmer). When performing the KatG expression analysis in response to C10 treatment, the amount of protein in each sample was measured by BCA (Pierce) and the amount of protein loaded in each lane was normalized to 67 ng to facilitate comparisons between samples.

### Purification of *Mtb* KatG protein

The *Mtb katG* coding region was cloned into *Not*I and *Eco*RI sites in the pGEX-6P-1 expression vector to translationally fuse glutathione-S-transferase to the N-terminus of the KatG protein and the expression of the fusion protein was induced in logarithmically growing *E. coli* BL21-DE3 cells by treating 1 L of cells with 0.1 mM IPTG for 4 h. Cells were pelleted, resuspended in 20 mL 1× PBS containing a 1× protease inhibitor cocktail, and lysed two times in a cell disruptor. Lysate was treated with 9 U/mL benzonase (Sigma) and clarified by centrifugation. GST-KatG was purified from the supernatant by incubating lysate overnight with glutathione agarose resin (Goldbio), washed with 300 mL 1× PBS, and eluted from the resin by cleaving the KatG protein from the GST tag using PreScission protease in buffer containing 50 mM Tris-HCl pH 7.0, 150 mM NaCl, 1 mM EDTA, and 1 mM DTT.

### Thermal shift assay on KatG protein

The melting temperature (*T*_*m*_) of KatG in the presence of C10 or INH was determined by differential scanning fluorimetry. Purified *Mtb* KatG protein (1.1 µM) was incubated with INH or C10 in 50 mM potassium phosphate buffer pH 7.0 containing 50 µM MnCl_2_ before mixing samples with SYPRO Orange (ThermoFisher) at a final concentration of 1× in a 96-well PCR plate. The plate was incubated for 5 s at increasing temperatures in 0.5°C increments from 10 to 95°C and fluorescence was monitored over time in the HEX channel on a CFX96 Real-Time System (Bio-Rad). The *T*_*m*_ was calculated by fitting curves with a Boltzmann sigmoidal equation in GraphPad Prism, and the Δ*T*_*m*_ was calculated as the difference between the *T*_*m*_ of each sample and the average of the untreated control samples.

### Hydrogen peroxide degradation assay

The catalase activity of either purified KatG or *Mtb* cell lysates was measured in a UV/Vis spectrophotometer using quartz cuvettes. For *in vitro* assays of purified KatG activity, 1.9 mL of 50 mM potassium phosphate buffer pH 7.0 containing 25 nM purified KatG was incubated with or without C10 and/or INH at 30°C for 5 min and the sample was used to blank the spectrophotometer before the indicated concentration of H_2_O_2_ was added to initiate the reaction, bringing the final volume of the reaction to 2 mL. For samples containing *Mtb* lysate, the samples were bead beat as described above and the protein concentration in the lysate was measured by BCA (Pierce). Samples were normalized such that 25 µg of total cell protein was present in each assay sample in 1.9 mL of 50 mM potassium phosphate buffer pH 7.0, and the reactions were initiated with the addition of H_2_O_2_ to a concentration of 5 mM in 2 mL final volume. The absorbance at 240 nm was read every 10 s for 2 min, and the negative slope of the curve was used to calculate the rate of H_2_O_2_ degradation. The absorbance was converted to molarity using the extinction coefficient for H_2_O_2_, *ε*_240_ = 43.6 M^−1^ cm^−1^.

### INH-activation assay and detection of INH-NAD in *Mtb*

The activation of INH was monitored *in vitro* using 200 nM purified KatG protein in 50 mM potassium phosphate buffer pH 7.0, 50 µM NAD^+^, 50 µM INH, 50 µM MnCl_2_, and the indicated concentration of C10 in a final volume of 1 mL. At the indicated time points, 100 µL was removed from the reaction and inactivated in 100 µL ice cold methanol and stored at −20°C before liquid chromatography/mass spectrometry (LC/MS). To monitor INH activation in live *Mtb*, 50 mL cultures of *Mtb* in Sauton’s media without Tween 80 were treated with the indicated concentration of C10 and/or INH for 3 days. To extract polar metabolites, the cultures were pelleted, washed twice in H_2_O, and resuspended in 1.5 mL of 2:1 chloroform:methanol in glass conicals. Samples were kept on ice and vortexed each for 1 min in 20-s intervals, and stored at 4°C overnight. Then 375 µL of H_2_O was added, the samples were vortexed for 1 min in 20-s intervals, keeping the samples on ice. Samples were incubated at room temperature for 1 h with constant agitation, and then centrifuged for 10 min at 1000 RPM. The top aqueous layer was transferred to a fresh 1.5 mL microcentrifuge tube, stored overnight at −20°C, centrifuged for 5 min to pellet any insoluble material, and the supernatant was transferred to a fresh tube. INH-NAD was detected using methods similar to those previously described (40, 59, 75) with some modifications. Ultra high-performance LC (UHPLC)/MS was performed with an Agilent 1290 Infinity UHPLC system interfaced with an Agilent 6530 QTOF mass spectrometer. Hydrophilic interaction liquid chromatography (HILIC) analysis was performed by using a HILICON iHILIC-(P) Classic column with the following specifications: 100 mm × 2.1 mm, 5 µm. Mobile-phase solvents were composed of *A* = 20 mM ammonium bicarbonate, 0.1% ammonium hydroxide (adjusted to pH 9.2) and 2.5 µM medronic acid in water:acetonitrile (95:5) and *B* = 2.5 µM medronic acid in acetonitrile:water (95:5). The column compartment was maintained at 45°C for all experiments. The following linear gradient was applied at a flow rate of 250 µL/min: 0–1 min: 90% B, 1–12 min: 90–35% B, 12–12.5 min: 35–20% B, and 12.5–14.5 min: 20% B. The column was re-equilibrated with 20 column volumes of 90% B. The injection volume was 2 µL for all experiments. Data were acquired in both positive and negative ion modes. The mass/charge (*m/z*) and retention times (RTs) of the compounds were as follows: INH *m/z* = 138.066188, RT = 1.87 min; C10 *m/z* = 378.11584, 0.92 min; NAD^+^ *m/z* = 664.116399, RT = 6.12 min; and INH-NAD *m/z* = 769.137863, RT = 5.70 min.

### Measurement of *de novo* lipid synthesis by ^14^C labeling and TLC

To monitor *de novo* mycolic acid biosynthesis, *Mtb* growing in Sauton’s liquid medium was adjusted to OD_600_ of 0.5, treated with the indicated concentrations of C10 and/or INH in 1 mL final volume, and immediately exposed to 2 µCi/mL ^14^C-acetate (PerkinElmer). After incubation at 37°C for 20 h, the cells were pelleted, and resuspended in 2:1 chloroform:methanol, vortexed, then samples were pelleted to remove insoluble cell debris, and the supernatant was transferred to a fresh vial. To separate lipid species by TLC, 40 µL of the sample was added dropwise to an HPTLC plate coated with silica gel 60 matrix (Sigma). TLC plates were developed in 75:10:1 chloroform:methanol:H_2_O and imaged by phosphorimaging on a Typhoon laser-scanner (Cytiva). The intensity of each band was quantified in ImageJ, normalized to the total intensity in the whole lane, and the fold change was quantified relative to the untreated control for each replicate. To assign putative identities to relevant bands, standards for ^14^C-acetate, TDM, free mycolic acid, and oleic acid were run on each plate. TDM purified from H37Ra (Invivogen) was derivatized to generate a free mycolic acid standard using methods similar to those previously described (76). Briefly, 100 µL of 0.5 mg/mL TDM in isopropanol was subjected to an alkaline ester hydrolysis by mixing with 2 µL H_2_O and 5 µL of 10M KOH, and heating to 90°C for 1 h to ensure efficient saponification. The resulting mycolic acids were purified from the trehalose by neutralizing the reaction with 50 µL 1.2M HCl, adding 100 µL chloroform, 100 µL H_2_O, vortexing, and separating the organic phase from the aqueous layer to obtain free mycolic acids in chloroform.

To monitor α-, methoxy-, and keto-mycolic acid biosynthesis, we adapted previously published methods (54, 77, 78), with some alterations. *Mtb* growing in Sauton’s liquid medium was adjusted to OD 0.5 and treated with the indicated concentrations of C10 or INH in 1 mL final volume of media containing 2 µCi/mL ^14^C-acetate. After incubation at 37°C for 48 h, cells were pelleted, resuspended in 1 mL H_2_O, transferred to 15 mL glass conicals containing 1 mL of 40% tetrabutylammonium hydroxide (Sigma), and then heated at 100°C overnight. This was followed by the addition of 2 mL of dichloromethane (Sigma) and 100 µL iodomethane (ThermoFisher) followed by constant mixing for 1 h. The upper aqueous phase was then discarded and the remaining liquid in the organic layer was allowed to evaporate overnight. The resulting mycolic acid methyl esters were resuspended in 1 mL diethyl ether (Sigma) and then transferred to 2 mL glass vials. Diethyl ether was then evaporated and resulting mycolic acid methyl esters were resuspended in 200 µL dichloromethane. Equal counts (2,500 CPM) were loaded onto TLC plates and developed three times in 85:15 petroleum ether:diethyl ether.

## Data Availability

Whole-genome sequencing data are deposited in the Sequence Read Archive (accession numbers PRJNA889365 and PRJNA1030020).

## SUPPLEMENTAL MATERIAL

Figure S1 mbio.02968-23-s0001.pdf The mutations in GHTB136 and GHTB146 impact the expression of neighboring genes.

Figure S2 mbio.02968-23-s0002.pdf *katG* mutants are not resistant to C10.

Figure S3 mbio.02968-23-s0003.pdf C10 does not increase KatG catalase activity or thermal stability.

Figure S4 mbio.02968-23-s0004.pdf C10 does not alter mycolic acid biosynthesis in the presence or absence of INH.

Table S1 mbio.02968-23-s0005.xlsx Full statistical analyses for data in main figures.

ASM does not own the copyrights to Supplemental Material that may be linked to, or accessed through, an article. The authors have granted ASM a non-exclusive, world-wide license to publish the Supplemental Material files. Please contact the corresponding author directly for reuse.
