# Supplementary material for: Inducing vulnerability to InhA inhibition restores isoniazid susceptibility in drug-resistant Mycobacterium tuberculosis
Source: mBio. 2024 Jan 31;15(3):e02968-23. doi: 10.1128/mbio.02968-23 (PMC10936210; doi:10.1128/mbio.02968-23)
Supplement: Figure S2 — katG mutants are not resistant to C10. [file mbio.02968-23-s0002.pdf]

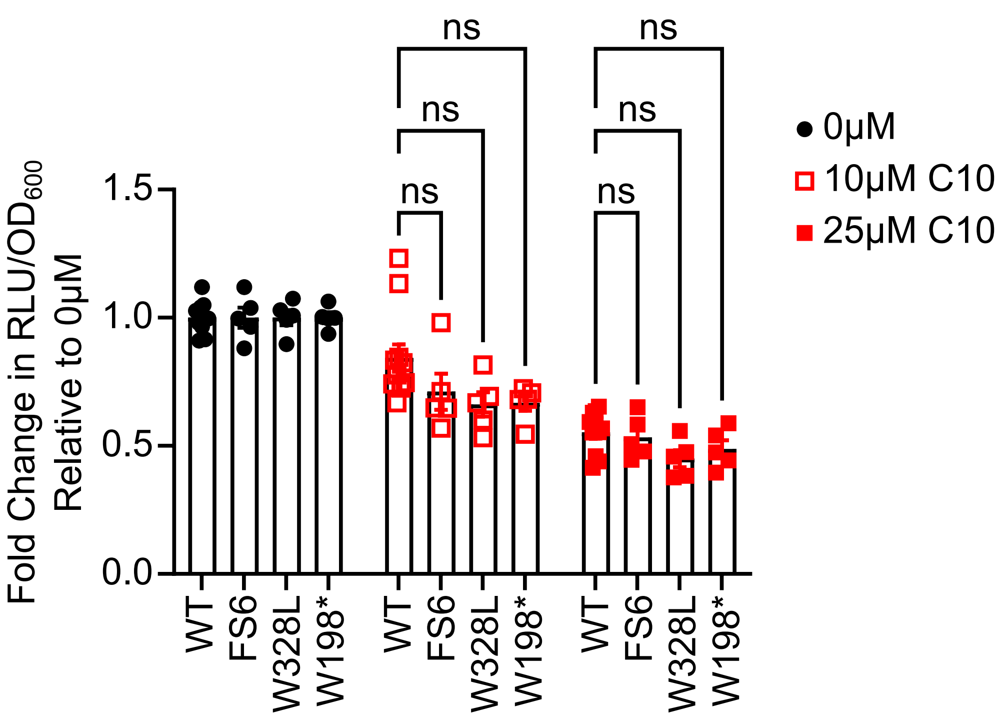

Figure S2: *katG* mutants are not resistant to C10. *Mtb* strains harboring the indicated *katG* alleles were cultured in Sauton's liquid medium containing 0, 10, or 25μM C10 for 24 hours and ATP levels were measured by the BacTiter Glo assay. The RLU were normalized to the OD_{600} of the culture to control for differences in cell density. The fold change in ATP levels was calculated relative to the 0μM C10 control for each strain, n=5-11. The data presented here is the same data present in Fig. 3F, reproduced here to highlight comparisons between each mutant at a given concentration of C10. A 1-way ANOVA with Tukey's post test was performed to identify statistically significant differences. ns, not significant.
