# Supplementary material for: Inducing vulnerability to InhA inhibition restores isoniazid susceptibility in drug-resistant Mycobacterium tuberculosis
Source: mBio. 2024 Jan 31;15(3):e02968-23. doi: 10.1128/mbio.02968-23 (PMC10936210; doi:10.1128/mbio.02968-23)
Supplement: Figure S3 — C10 does not increase KatG catalase activity or thermal stability. [file mbio.02968-23-s0003.pdf]

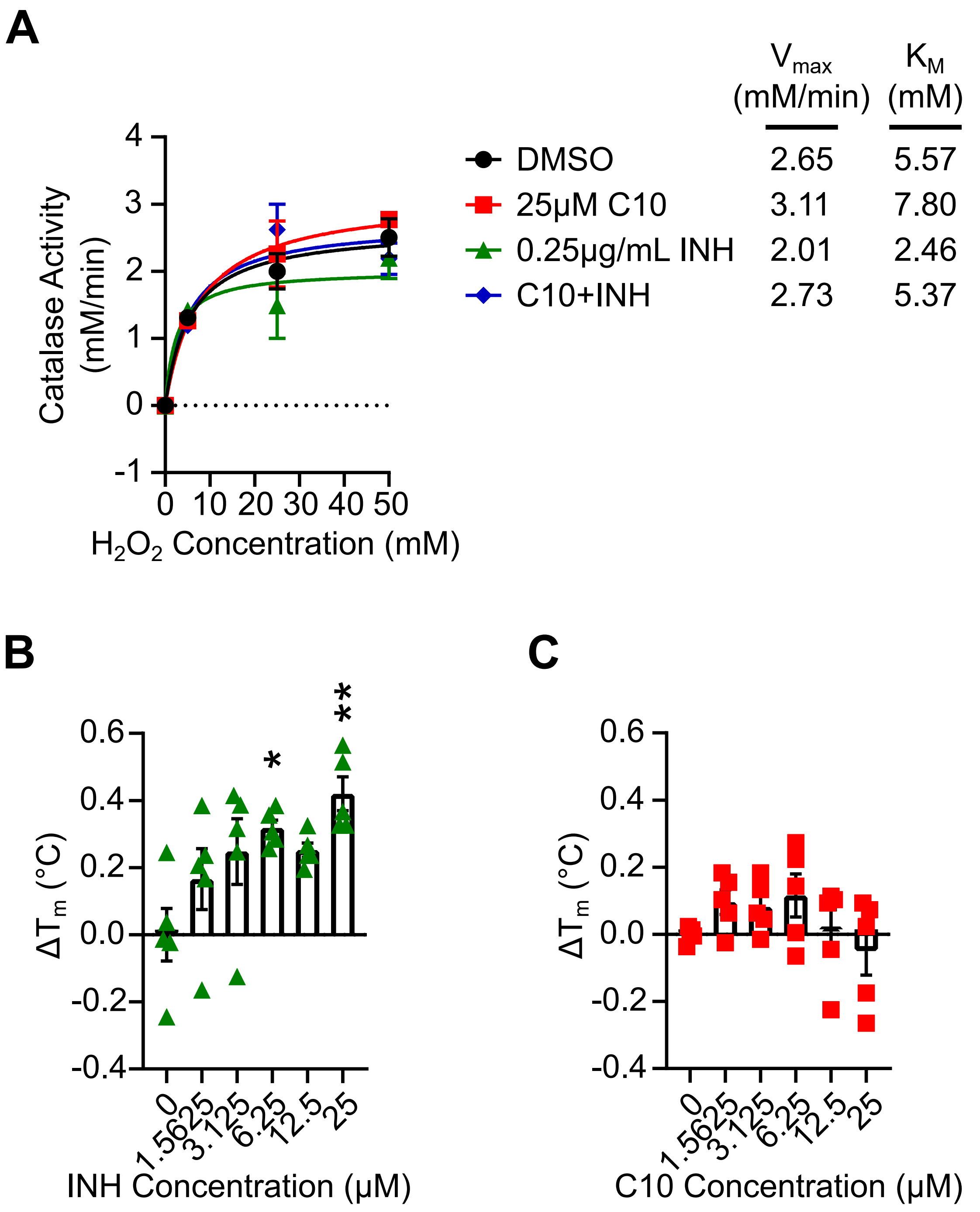

Figure S3: C10 does not increase KatG catalase activity or thermal stability. (A) Purified *Mtb* KatG protein (25nM) was incubated with or without 25μM C10 and/or 0.25μg/mL INH in 50mM potassium phosphate buffer pH 7.0, and the catalase activity, or H_2O_2 degradation, was monitored through a decrease in absorbance over time at λ_{abs}=240nm in a UV/Vis spectrophotometer, n=3. The data in the graph was fitted with the Michaelis-Menten equation in GraphPad Prism to determine the V_{max} and K_M of KatG in each condition, which are indicated in the figure legend. (B-C) Purified *Mtb* KatG protein (1.1μM) was incubated with (B) INH or (C) C10 in 50mM potassium phosphate buffer pH 7.0 with 50μM $MnCl_2$ before mixing samples with Sypro Orange, incubating at increasing temperatures, and monitoring fluorescence over time. The melting temperature (T_m) was calculated by fitting curves with a Boltzmann sigmoidal equation in GraphPad Prism, and the ΔT_m was calculated as the difference between the T_m of each sample and the average of the untreated control samples (n=5). Statistically significant differences were identified by performing a 1-way ANOVA with Tukey's post test, and data points that are statistically significantly different from the untreated control are indicated with stars. * P<0.05, ** P<0.01.
