# Supplementary material for: Inducing vulnerability to InhA inhibition restores isoniazid susceptibility in drug-resistant Mycobacterium tuberculosis
Source: mBio. 2024 Jan 31;15(3):e02968-23. doi: 10.1128/mbio.02968-23 (PMC10936210; doi:10.1128/mbio.02968-23)
Supplement: Figure S4 — C10 does not alter mycolic acid biosynthesis in the presence or absence of INH. [file mbio.02968-23-s0004.pdf]

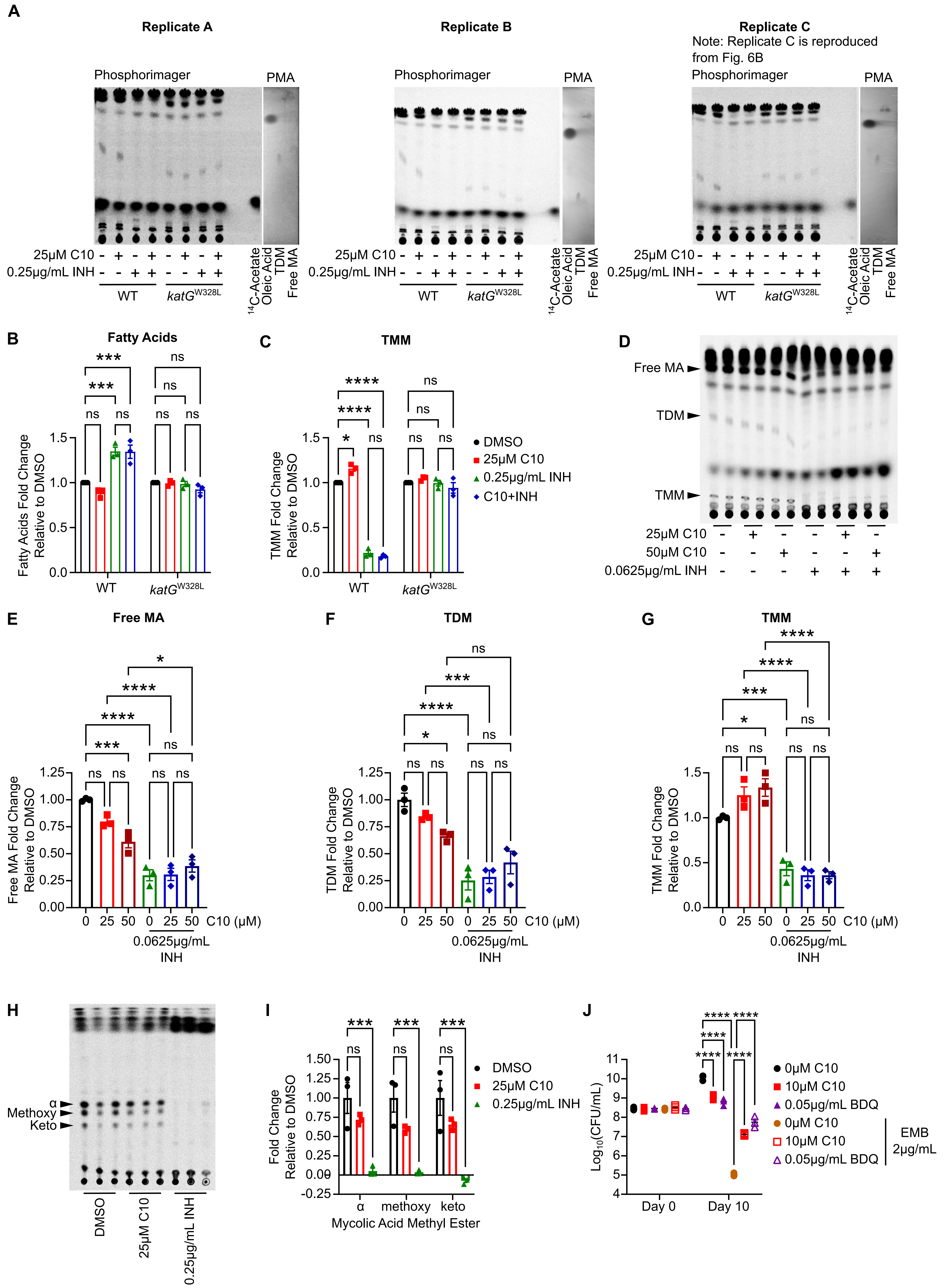

Figure S4: C10 does not alter mycolic acid biosynthesis in the presence or absence of INH. (A) WT or *katG*^{W328L} *Mtb* was cultured in Sauton liquid medium, treated with 25μM C10 and/or 0.25μg/mL INH, and immediately exposed to 2μCi/mL of ^{14}C-labeled acetate. After 20 hours, lipids were extracted and analyzed by TLC to measure the *de novo* synthesis of mycolic acids and other lipids. The TLC plate was developed with 75:10:1 Chloroform:Methanol:H_2O and radioactivity was analyzed by phosphorimaging. Bands corresponding to free mycolic acid (MA), fatty acid, trehalose dimycolate (TDM), and ^{14}C-acetate were identified by comigration with a standard. Cold standards were imaged by coating the TLC plate in phosphomolybdic acid (PMA) and charring. Note that trehalose monomycolate (TMM) is putatively identified and not correlated with a standard. (B-C) The intensity of bands corresponding to (B) TMM and (C) fatty acids were quantified in ImageJ, and normalized to the DMSO sample, with each WT sample being normalized to WT DMSO and each *katG*^{W328L} being normalized to *katG*^{W328L} DMSO in order to compare across replicates, n=3. Note that one of the images in panel A, is the same image that is presented in Figure 6 in the main text, reproduced here for comparison to the other replicates. (D-G) WT *Mtb* was cultured in Sauton's liquid medium, treated with 25 or 50μM C10 and/or 0.0625μg/mL INH and immediately exposed to 2μCi/mL of ^{14}C-labeled acetate for 20 hours before lipids were extracted and analyzed by TLC using the same conditions as panel A. (E-G) The bands corresponding to (E) free MA, (F) TDM, and (G) TMM were quantified in Image J and normalized to the DMSO control to facilitate combining replicates from separate TLC plates. (H-I) WT *Mtb* was treated with 25μM C10 or 0.25μg/mL INH and immediately exposed to 2μCi/mL of ^{14}C-labeled acetate for 48 hours. Mycolic acid methyl esters were prepared and the TLC plate was developed 3 times with 85:15 petroleum ether:diethyl ether before radioactivity was analyzed by phosphorimaging. (H) An image of the plate is depicted, and (I) the intensity of bands in corresponding to α-, methoxy-, and keto-mycolic acids in panel H were quantified in ImageJ, and normalized to the DMSO sample, n=3. (J) WT *Mtb* was cultured in Sauton's liquid medium with the indicated concentrations of C10, BDQ, and/or EMB, and CFU/mL were enumerated on day 0 and 10 of treatment to determine the number of viable bacteria in each sample, n=3. Statistically significant differences were determined by 2-way ANOVA with Tukey's post test in panels B-C and I-J and 1-way ANOVA with Tukey's post test in panels E-G and select pairwise comparisons are depicted in the figure. ns not significant, * P<0.05, *** P<0.001, **** P<0.0001.
