# Supplementary material for: Inducing vulnerability to InhA inhibition restores isoniazid susceptibility in drug-resistant Mycobacterium tuberculosis
Source: mBio. 2024 Jan 31;15(3):e02968-23. doi: 10.1128/mbio.02968-23 (PMC10936210; doi:10.1128/mbio.02968-23)
Supplement: Figure S1 — The mutations in GHTB136 and GHTB146 impact the expression of neighboring genes. [file mbio.02968-23-s0001.pdf]

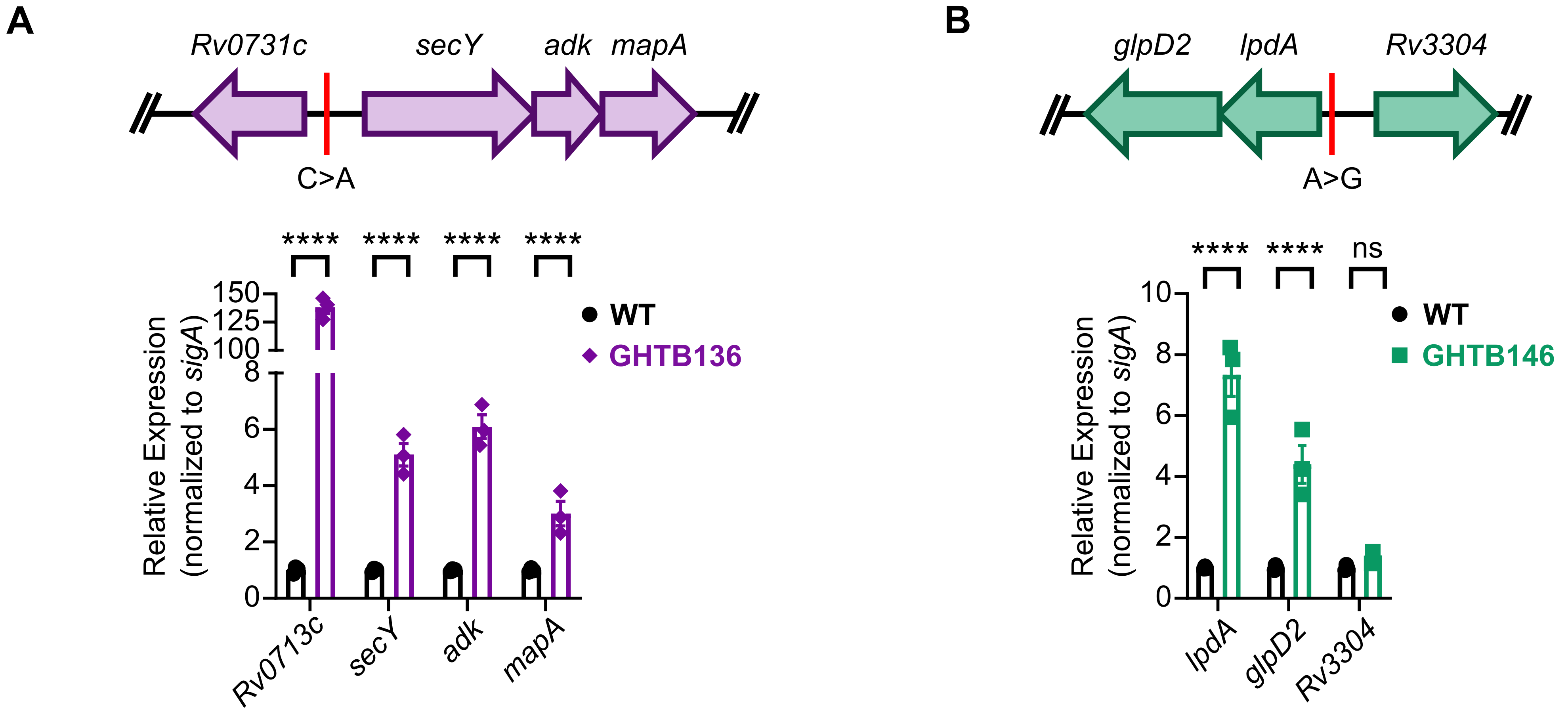

C

Gene	Function	References
Rv0731c	(Hypothetical) putative SAM-dependent methyltranferase	Uniprot
secY	Integral membrane protein, forms part of core Sec translocon channel	Brundage 1990 - *E. coli*
adk	Adenylate kinase (ATP:AMP Phosphotransferase), reversibly catalyzes ATP+AMP = 2ADP	Munier-Lehmann 1999
mapA	Methionine aminopeptidase, hydrolyzes N-terminal Met residue during translation, not essential due to presence of primary enzyme MapB	Vanunu 2019
lpdA	While mis-named as lipoamide dehydrogenase, actually catalyzes NAD(P)H dehydrogenase reaction NAD(P)H+Quinone = NAD(P)$^+$+Quinol	Argyrou 2004
glpD2	(Hypothetical) putative glycerol-3-phosphate dehydrogenase, catalyzing glycerol-3-phosphate+Quinone = dihydroxyacetone-phosphate+Quinol	Uniprot
Rv0830	(Hypothetical) putative SAM-dependent methyltranferase	Uniprot

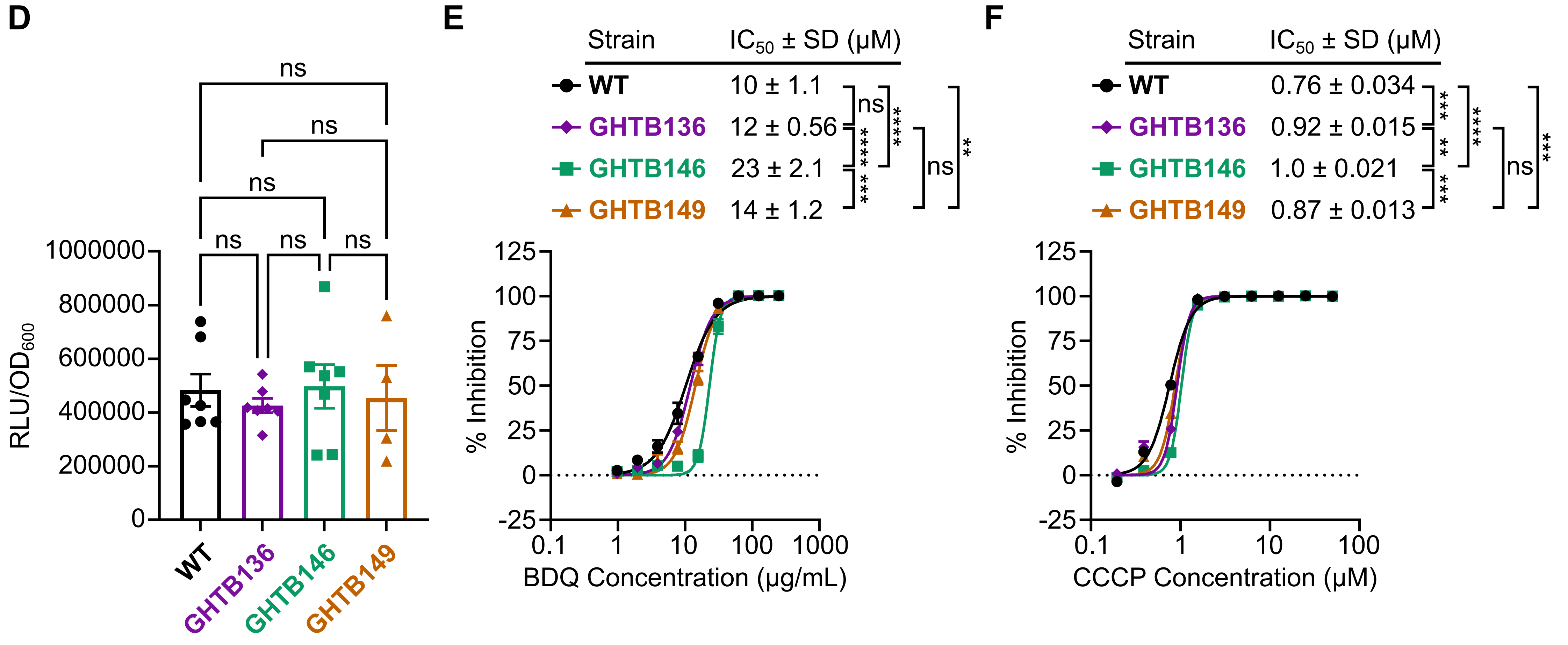

Figure S1: The mutations in GHTB136 and GHTB146 impact the expression of neighboring genes. (A-B) The expression of the indicated genes was monitored in (A) GHTB136 or (B) GHTB146 compared to WT *Mtb* by qRT-PCR, n=3. A map of the gene locus as well as the mutation present in the respective mutant strain is depicted above each graph. The data was log-transformed before performing a 2-way ANOVA with Tukey's post test to identify statistically significant differences between samples. (C) A table of the genes affected in GHTB136, GHTB146, and GHTB149 and their known functions. Functions are designated hypothetical if no studies could be found that validated the predicted function of the indicated gene. (D) The indicated strain of *Mtb* was cultured in Sauton's liquid medium and ATP levels were measured by the BacTiter Glo assay. Data was collected from either 2 (GHTB149) or 3 (WT, GHTB136, GHTB146) independent experiments performed on separate days, n=4-7. The relative luminescence units (RLU) were normalized to the OD_{600} of the culture to control for differences in cell density. A one-way ANOVA with Tukey's post test was performed to determine if any of the strains had significantly different levels of ATP at baseline. ns, not significant. (E-F) The indicated strain of *Mtb* was cultured in the presence of increasing concentrations of (E) BDQ or (F) CCCP for 1 week, and the % inhibition of *Mtb* growth and metabolism was determined using the resazurin assay, n=3. A best fit curve was determined in GraphPad Prism. IC_{50} values were log-transformed before performing a 1-way ANOVA with Tukey's post test to identify statistically significant differences. ns not significant, ** P<0.01, *** P<0.001, **** P<0.0001
